# Distance in cancer gene expression from stem cells predicts patient survival

**DOI:** 10.1371/journal.pone.0173589

**Published:** 2017-03-23

**Authors:** Markus Riester, Hua-Jun Wu, Ahmet Zehir, Mithat Gönen, Andre L. Moreira, Robert J. Downey, Franziska Michor

**Affiliations:** 1 Department of Biostatistics and Computational Biology, Dana-Farber Cancer Institute, and Department of Biostatistics, Harvard School of Public Health, Boston, MA, United States of America; 2 Cell Biology Program, Memorial Sloan Kettering Cancer Center, New York, NY United States of America; 3 Department of Epidemiology and Biostatistics, Memorial Sloan Kettering Cancer Center, New York, NY United States of America; 4 Department of Pathology, Memorial Sloan Kettering Cancer Center, New York, NY United States of America; 5 Thoracic Service, Department of Surgery, Memorial Sloan Kettering Cancer Center, New York, NY United States of America; University of Tennessee Health Science Center, UNITED STATES

## Abstract

The degree of histologic cellular differentiation of a cancer has been associated with prognosis but is subjectively assessed. We hypothesized that information about tumor differentiation of individual cancers could be derived objectively from cancer gene expression data, and would allow creation of a cancer phylogenetic framework that would correlate with clinical, histologic and molecular characteristics of the cancers, as well as predict prognosis. Here we utilized mRNA expression data from 4,413 patient samples with 7 diverse cancer histologies to explore the utility of ordering samples by their distance in gene expression from that of stem cells. A differentiation baseline was obtained by including expression data of human embryonic stem cells (hESC) and human mesenchymal stem cells (hMSC) for solid tumors, and of hESC and CD34+ cells for liquid tumors. We found that the correlation distance (the degree of similarity) between the gene expression profile of a tumor sample and that of stem cells orients cancers in a clinically coherent fashion. For all histologies analyzed (including carcinomas, sarcomas, and hematologic malignancies), patients with cancers with gene expression patterns most similar to that of stem cells had poorer overall survival. We also found that the genes in all undifferentiated cancers of diverse histologies that were most differentially expressed were associated with up-regulation of specific oncogenes and down-regulation of specific tumor suppressor genes. Thus, a stem cell-oriented phylogeny of cancers allows for the derivation of a novel cancer gene expression signature found in all undifferentiated forms of diverse cancer histologies, that is competitive in predicting overall survival in cancer patients compared to previously published prediction models, and is coherent in that gene expression was associated with up-regulation of specific oncogenes and down-regulation of specific tumor suppressor genes associated with regulation of the multicellular state.

## Introduction

Signatures based upon the expression levels of subgroups of genes in tumor samples have been explored in an effort to classify tumors and to predict the likelihood of survival of cancer patients [1–6]. These signatures are usually determined by identifying the subset of differentially expressed genes that stratify a patient cohort of a given histology into those with short versus long survival times (e.g. [2–5]). Despite being prognostic for the data sets from which they were derived, few such signatures have been able to be validated in independent patient cohorts [1, 6]. A significant limitation of this approach is that signatures need to be identified for each histologic type, as the prognostic benefit of a signature for one cancer type contains very little information about another. It is thus an important goal of the field to identify gene expression-based approaches that reliably predict patient survival for any tumor type.

We hypothesized that the distance of a tumor sample in gene expression from that of stem cells contains information about differentiation that can be extracted for, among other things, prediction of survival of a patient with any tumor type. We designed a novel methodology based on determining the distance of a cancer specimen's gene expression from that of undifferentiated cells, such as human embryonic stem cells (hESC). Our methodology is based upon the premise that histopathological classification of tumors relies on the differentiation status of tumor cells [7], and information about differentiation encoded in a tumor’s gene expression profile can be utilized for the objective prediction of patient survival for any tumor type. Our goal is to provide a method that can be applied to all cancer types regardless of availability of data on tissue-specific stem cells. We have therefore not investigated an exhaustive set of stem cell datasets.

Prior work by other researchers has attempted to compare a cancer’s gene expression to that of stem cells, either by identifying significantly differentially expressed genes in poor prognosis cancers and investigating if a subset of these have been associated with stem cell expression [8], or by identifying a limited list of genes associated with the stem cell phenotype, and seeing if this list is differentially expressed in poor prognosis cancers [9]. Our approach represents a significant advance over these prior published approaches, in that, it allows comparison of the more than 20,000 genes assayed in a gene expression array between the expression of cancers of any histology (i.e. carcinomas, sarcomas, and hematopoietic) and of normal stem cells.

## Methods

After a Waiver of Authorization and approval to perform this study was received from the Memorial Sloan Kettering Cancer Center Institutional Review Board, a retrospective review of patient medical records was performed. An overview of training (‘tuning’) and validation datasets of all cancers analyzed in this study is provided in Table A in the S1 File, with preprocessing and other details provided in the S1 File. The complete analysis is available as annotated Sweave/R code at https://bitbucket.org/lima1/scpaper.

### Three tuning parameters of the stem cell distance model

Our model contains three parameters to calculate the distance in expression between stem cell samples and patient samples used for survival prediction. Here, ‘parameters’ refer to tunable variables which control (i) the gene filter, in order to choose a cutoff of genes included in the determination of differences in expression between stem cells and cancer samples, (ii) the distance metric, in order to determine the distance in gene expression between stem cells and cancer samples, and (iii) the choice of stem cell expression data used for calculating the stem cell distance. Once having determined the stem cell distances for every patient sample, the potential of these distances in prediction of survival or recurrence was evaluated with a univariate Cox proportional hazards model. As outlined below, we tested the dependence of the parameter choice on the correlation of stem cell distance and overall or recurrence-free survival.

The first parameter controls the gene filter. We defined the filter over the interquartile range (IQR), a commonly used filter [10] in microarray studies. This choice was made because with appropriate cutoffs, it removes genes with expression changes that are thought to be too small to be biologically relevant. These genes are normally removed to increase sensitivity when controlling for multiple testing. Here, the main purpose of the gene filter is to remove noise from the stem cell distance calculation. The gene filter is defined as:

$$F(g_{i})=\left\{ \begin{array}{l} 0\;if\;\max\left(g_{i}\right)<{log}_{2}^{}(100),\\ \;1\;else\;if\;\mathrm{IQR}\left(g_{i}\right)\geq c∙median(IQR(g)),\\ \;0\;else. \end{array}\right.$$

Here IQR(g_i_) is the interquartile range of gene *i*. To make the cutoff more intuitive and comparable across datasets, we defined it as a product of a tuning parameter *c* and the median IQR over all genes (IQR(g)). For example, c = 1 removes half of the probe sets, those with the lowest IQR. A value of F(g_i_) = 0 results in gene *i* being removed from the analysis. The log_2_(100) filter removes genes with consistently low expression, before applying the IQR cutoff. The parameter *c* was analyzed in a grid search, corresponding to retaining the top 5, 10,…, 95% of all probe sets.

The second parameter determines the distance metric. We considered the Pearson Correlation distance (1 – Pearson Correlation Coefficient) and the Euclidean distance. Both metrics displayed good performances in a previous study [11], with the Pearson Correlation distance achieving slightly better results than the Euclidean distance. Expression values for stem cell samples were averaged over 3 hESC samples (GSM176743, GSM176747, GSM176752) to determine average hESC expression, 3 hMSC samples (GSM176732, GSM176734, GSM176738) to determine average hMSC expression, and 10 CD34+ cell samples (GSM240500-GSM240509, GSE30377) to determine average expression of primitive hematopoietic cells. We also tested hematopoietic progenitors derived from bone marrow (BM), cord blood (CB) and peripheral blood (PB). Distances were then calculated from the centroids to the patient samples.

### Error estimation and parameter identification

For model tuning, we estimated the prediction accuracy of the Cox model with the concordance probability estimate (CPE [12]). Several methods for concordance estimation have been developed for censored data (e.g., [12, 13]). We used the CPE [12] as concordance estimator because it was utilized in the Director’s Challenge (DC) study [14]. Since the different concordance metrics mainly differ in the way they deal with censoring and since our datasets were large, we obtained highly similar models when optimizing different metrics and we thus only used the CPE throughout our work.

For each parameter combination, the stem cell distance was calculated and the error in predicting survival was evaluated by 5-fold cross-validation, which was repeated 100 times with different random folds to obtain stable error estimates. This procedure thus resulted in one score per parameter combination after averaging the 100 prediction error estimates.

We performed the parameter search as described above, respectively, on ‘tuning data’ (denoted as training datasets in Table A in S1 File) of lung adenocarcinoma, breast cancer, liposarcoma, colorectal cancer, ovarian cancer, acute myeloid leukemia (AML), and diffuse large B-cell lymphoma (DLBCL) samples to identify optimal parameter combination based on achieving highest mean CPE scores across 100 5-fold cross-validations, and then tested the models in the independent datasets (denoted as not training datasets in Table A in S1 File) for all above cancer types (Figs A-G and Table A in S1 File). Note that parameter tuning generates highly correlated models; model over-fitting is therefore less likely than in multivariable training in which features can be combined until the model perfectly explains the response data. However, to avoid reporting over-optimistic estimates, all results refer to estimates obtained in data not used for tuning unless stated otherwise.

### Published classifiers

For adenocarcinoma of the lung, we compared our predictor to several published predictors or ‘gene signatures’ [14–17]; These are all the predictors or gene signatures published in the Director’s Challenge (DC) study [14] and in studies citing the DC study. We reproduced all gene signature-based classifiers and compared them to their published results. Other classifiers, such as classifier A from [14], use stochastic optimization algorithms and we urge authors of such methods to publish their code and seeds in order to address reproducibility issues of a nondeterministic feature selection. For classifiers we could not reproduce, we only report the published performance. The reproduced classifiers were also trained on the UM/HLM training dataset. As in the DC study [14], the versions of classifiers using clinical covariates incorporated these in a ridged regression. For breast cancer, we obtained risk scores from the van’t Veer signature [2] and from Gene expression Grade Index (GGI) [3] model. For both of these models, we used the implementation in the genefu Bioconductor package (gene70/ggi functions). Also as in [14], all risk scores were quantile normalized, so that the risk scores of all predictors had an IQR of 1.0 and a mean of 0.0. This approach allowed for a comparison of predictors by risk score hazard ratios. Continuous predictors were stratified based on risk score tertiles into low-, medium- and high-risk groups.

### New stem cell signatures derived from hESC distances

We next generated a parsimonious predictor based on our hESC distance. For each gene, we estimated the overall association of expression with hESC distance across all cohorts for breast and lung, the two histologies for which the hESC distance had strongest association with survival compared to other stem cells. Genes with variance of 0 in any dataset were removed. For all other genes, the hESC distance was modeled as a function of gene expression. The overall regression coefficient for each gene was then pooled by a fixed-effects model, in which the cohort regression coefficients were weighted according to the inverse of their standard errors. This meta-analysis was performed with the metafor R package. The top 500 genes associated with hESC distance represented our hESC gene signature. This approach was repeated for the hMSC distance in all remaining solid tumors and for the CD34+ distance in hematological malignancies. The large signature size was chosen to obtain enough statistical power for pathway analyses. For survival prediction, we only used the top 200 genes, as described recently [18]. In short, genes and their expression values were weighted by their pooled regression coefficient to calculate a risk score, i.e. expression values of genes positively correlated with hESC distance were added to the risk score, while expression values of negatively correlated genes were subtracted.

The performance of our signatures were then compared with another signature of genes associated with hESC expression [9] (Fig H (a)-(d) in S1 File). These genes were shown to be prognostic in lung adenocarcinoma [19]. A mapping of gene symbols to Affymetrix probe ids of these genes was obtained from the supplementary material of Hassan et al. [19]. For AML and DLBCL, we compared the performance of our CD34+ signature to a hematopoietic stem cell signature [20] (Figs H (e)-(f) in S1 File), which was shown to be prognostic in AML. Probe sets were obtained from the supplement of that paper.

### Hierarchical cluster analysis

As we have shown previously [11], phylogenetic methods can be used to construct lineages of tumor subtypes. Here we used a similar approach that clusters individual patients as in classic hierarchical clustering but, in addition, displays the distances to stem cells. The cluster dendrogram was constructed with FastME [21] and implemented in the ape R package [22]. The dendrogram was visualized with Dendroscope 2.7.4 [23]. FastME was chosen over other non-likelihood phylogenetic methods (e.g., Maximum Parsimony, Neighbor-Joining or Weighted Least Squares) because of its computational efficiency and proved accuracy when applied to microarray data [11, 24]. The lack of likelihood models for gene expression changes currently prohibits the use of likelihood or Bayesian phylogenetic methods.

### Biological enrichment analysis

DAVID [25] (http://david.abcc.ncifcrf.gov/) was used to analyze biological enrichment of the probe sets that displayed a high correlation with stem cells. In order to obtain a list of all gene sets and pathways, we obtained a DAVID functional annotation chart report and visualized it with the Cytoscape 2.8.2 plug-in Enrichment Map (v1.2) [26]. The parameters for the enrichment map were: p-value cutoff: 0.005, FDR Q-value cutoff: 0.1, and Jaccard coefficient: 0.25. A DAVID enrichment chart belonging to the Ben-Porath signature was used as enrichment set 2. Ingenuity Pathways Analysis (Ingenuity® Systems, www.ingenuity.com) was used to infer transcription factor activation/inhibition status.

## Results

### Expression datasets and parameter identification

We first focused our study on the two most common cancer types (breast and lung) and then demonstrated the generalizability of this approach in 5 other cancer histologies. In total, mRNA expression data from 4,413 patient samples of 20 individual datasets (Table A in S1 File) were used to explore the utility of ordering samples by their distance in gene expression from that of stem cells. A differentiation baseline was obtained by including expression data of human embryonic stem cells (hESC) [27] and human mesenchymal stem cells (hMSC) [27] for solid tumors, and of hESC and CD34+ cells [28] for liquid tumors (see Supporting Information for details of these datasets). The hESC samples are a mixture of H1 male and H9 female hESCs. As described in Table A in S1 File, 7 datasets were used to tune the stem cell distance based predictor, and the 13 left out datasets were used to test the predictor. Parameter search was performed by evaluating association with survival outcome (Fig A in S1 File, described in Methods). Strikingly, in most analyzed tumors, the optimal parameters were very similar. Prediction accuracies close to the optimum were achieved when using the Pearson Correlation distance and a gene filter that retains all genes with IQR larger than twice the median IQR. This cutoff thus represented a robust compromise between removing noise and retaining signal, and can be used for other cancer types without training with survival data. Therefore, this method can be applied on regular datasets through model training and is also promising in some cases with very limited sample size by using the broadly applicable parameters. All results in this paper were obtained using these default parameters.

### Stem cell distance associated with clinical data

**Fig 1:**
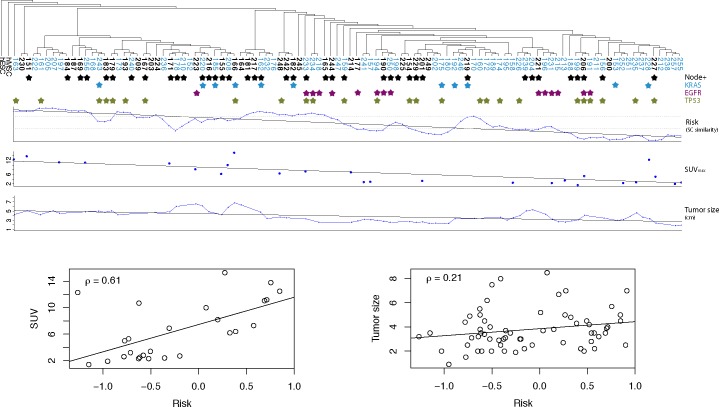
Clustering of the lung adenocarcinoma validation dataset (MSK cohort). Patient samples are clustered based on their distances of gene expression profiles from stem cells (see hierarchical cluster analysis section of the Methods). Samples marked with a bold, black label indicate deceased patients. Plotted below the dendrogram are lymph node involvement (node-negative versus node-positive) and the presence of KRAS, EGFR and/or TP53 mutations. Stars indicate the patients with positive lymph nodes test results or corresponding gene mutations. Furthermore, we show the patient risk scores, obtained by a Cox proportional hazards model using the distance to hESC as covariate. This model was fitted in the UM/HLM training set. Dotted grey lines indicate the risk score tertiles in the training cohort. A subset of patients had FDG-PET imaging prior to treatment. The SUV_max_ describes the maximal measured glucose uptake of the tumors, and is plotted below the risk score. Risk score and SUV_max_ were highly correlated (ρ = -0.613, P < 0.001). Finally, the size of the tumor was plotted for the 63 patients for whom this information was available (ρ = -0.209, P = 0.1). Curves of risk score and tumor size were smoothed with a 3-point simple moving average (SMA).

Tumors were first stratified based on their distance in expression from that of stem cells into 3 equally sized groups. Given the known association between the prognosis of a lung cancer and clinical variables such as histologic differentiation, stage (tumor size and presence or absence of nodal metastases), and ^18^F-flouro-deoxyglucose (FDG) avidity imaged with positron emission tomography (PET), we first examined whether the distance from stem cell gene expression of lung cancers correlated with prognostic histologic and clinical variables in a clinically coherent manner (Fig 1). For the Director’s Challenge [14] validation cohort from the Memorial Sloan-Kettering Cancer Center (‘MSK dataset’), extensive clinical and demographic data was available (Table B in S1 File). Limited similar clinical data was also available for the breast cancer dataset (Table C in S1 File). We found that the majority of histologically poorly differentiated tumors demonstrated expression similar to stem cells (lung adenocarcinoma: P < 0.001; breast cancer: P < 0.001, Fisher’s exact test; Tables B and C in S1 File). In the lung adenocarcinoma specimens, similarity to stem cell expression was significantly associated with the presence of nodal metastases (P < 0.001), stage (P < 0.001) and pre-operative FDG-PET maximal standard uptake values (SUV_max_), a measure of increased tumor glucose uptake shown to be prognostic [29] (δ = 0.613, P < 0.001) (Fig 1). We observed that female (P < 0.001) and never-smoker (P = 0.004) lung cancer patients (two groups who more often have better prognosis) were over-represented in the group with expression furthest from stem cells. Finally, the distribution of DNA mutations (*KRAS*, *EGFR*, and *TP53*) was not significantly associated with distance from stem cell expression.

### Stem cell distance predicts patient survival

**Fig 2:**
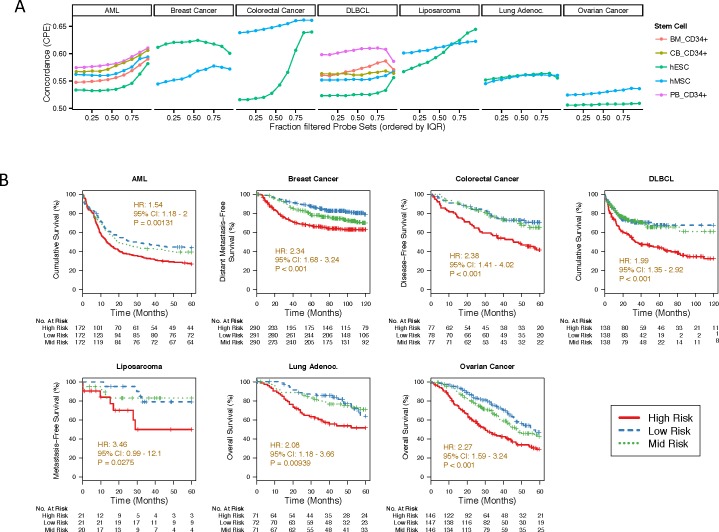
Survival analysis of the stem cell distance-based risk predictor. **(A)** Dependence of the prediction concordance of the choice of the stem cell dataset and the variance probe set filter, the two main parameters of the model, in all tuning datasets. The concordance is given on the y-axis as the concordance probability estimates (CPEs), with a value of 0.5 indicating a random model, and a value of 1.0 a perfect model. (**B**) Kaplan-Meier plots for all analyzed cancer types, visualizing survival differences among three risk groups. Samples of all validation cohorts were trisected into three equally sized groups based on their expression distances to stem cells. The high-risk group represents the samples close to the stem cells, and the low-risk group represents the samples farthest from stem cells. Validation cohorts were then combined for each cancer type (see Figs B-G in S1 File for Kaplan-Meier plots for all cohorts, including tuning datasets, separately). Note that the distance in gene expression of a sample from that of stem cells is a continuous measure; the subdivision of samples was chosen only to visualize the differences in survival between these groups. For all cancer types, data from validation cohorts (Table A in S1 File) is used for the analysis. The stem cell signatures used are PB_CD34 for AML and DLBCL, hESC for breast cancer and lung cancer, hMSC for colorectal cancer, liposarcoma and ovarian cancer (Table M in S1 File). Hazard ratios and 95% confidence intervals of normalized risk scores are shown. P values were calculated with the log-rank test.

We then applied our computational methodology to multiple diverse cancer histologies including other epithelial (colon and ovary), mesenchymal (liposarcoma), and hematopoietic (lymphoma and leukemia) malignancies. In each dataset, we found that patients whose samples displayed a gene expression pattern closest to that of stem cells experienced significantly worse survival compared to patients with expression farthest from stem cells (Fig 2 and Figs D-G in S1 File). We first investigated the concordance of our predictor, i.e. the probability that of a random pair of patients, the patient with the higher estimated risk had the poorer outcome. In Fig 2A, we summarize the parameter tuning by showing the influence of the two most important parameters, the gene filter and the choice of the stem cell data, on the prediction performance in the tuning data. The highest concordance was typically achieved when the 75% of genes with lowest variance were removed. In liquid tumors, CD34+ cells achieved highest concordance; hESC was superior in lung adenocarcinoma and breast cancer. In the remaining histologies, the hMSC distance was best predictive of survival. We then again stratified all samples from all validation datasets into three equally sized risk groups based on their distance to stem cells (Fig 2B). In all tumor types, the high-risk group had statistically significantly shorter survival than the low-risk group.

### Comparison between stem cell distance and other methods

**Fig 3:**
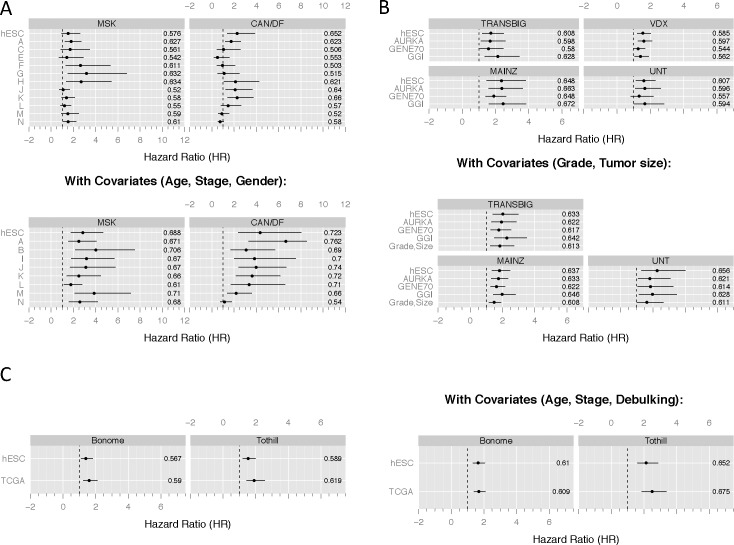
Prognostic power of the stem cell distance-based risk predictor. **(A-B)** Classifier performance of novel and published classifiers in lung adenocarcinoma **(A)** and breast cancer **(B)** validation cohorts. All risk scores were quantile normalized, so that the risk scores of all predictors had an IQR of 1 and a mean of 0. This approach allowed for a comparison of predictors by risk score hazard ratios. Hazard ratios and 95% confidence intervals of normalized risk scores for the stem cell distance-based predictor (SC) as well as for competing predictors are shown. A hazard ratio significantly larger than 1 indicates that patients with a high predicted risk had a poor outcome. Numbers on the right of the each row of plots represent the CPEs. Shown on the top are the results of a model using only gene expression information; at the bottom, we show the results of a multivariate model in which clinical covariates were incorporated. **(A)** Classifiers A-N are the published results of the mostly gene signature-based Director's Challenge predictors [14]. **(B)** The AURKA prediction is obtained by a univariate model using only the expression of the *AURKA* gene as covariate. The model GENE70 represents the prediction of the van't Veer gene signature [2] comprising 70 genes. The GGI prediction represents the Gene expression Grade Index [3]. Tumor size was not available in the VDX cohort. **(C)** Comparison in high grade, serous ovarian cancer with the survival signature published by the TCGA project. See the Supporting Information on details of the datasets and methodology.

We then compared the predictive accuracy of our stem cell distance-based predictor with the performance of published signatures for lung adenocarcinoma and breast cancer, for which multiple datasets were available. For lung adenocarcinoma, we investigated the gene-signature based classifiers from the Director’s Challenge study [14] (Fig 3A and Tables D-G in S1 File). For breast cancer, we investigated the performance of our predictor relative to a univariate model using the expression of the *AURKA* gene as covariate, since this model serves as a robust benchmark for other breast cancer predictors [30], and two validated gene signature-based predictors [2, 3] (Fig 3B and Tables H-I in S1 File). In ovarian cancer, we compared the stem cell distance with the gene signature developed by the TCGA project, which recently was identified as best prognostic model in high grade, serous ovarian cancer [18, 31] (Fig 3C and Tables J-K in S1 File). Compared to all other classifiers, our stem cell distance-based predictor displayed a robust performance in both cancer types, in all validation sets, and with and without the use of clinical covariates (Fig 3A–3C and Tables D-J in S1 File). While most previously published predictors failed in some of the validation sets, our predictor was consistently among the top ranking predictors, always achieving hazard ratios statistically significantly higher than 1, i.e. an increase in stem cell similarity was always associated with a higher risk of an event. For all datasets, we collected all available clinical characteristics associated with outcome and stem cell distance consistently provided additional prognostic information, with the exception of AML (Table L in S1 File).

### Different performance achieved in different tumor subtypes

We then stratified the breast cancer samples by subtypes (ER+/HER2-, ER-/HER2-, HER2+) and combined samples belonging to each subtype from all datasets. By analyzing the stratified risk scores (S3F–S3H Fig in S1 File), we found that the stem cell distance-based predictor displayed a good performance in ER+/HER2- (HR 2.14; 95% CI, 1.46 to 3.13; P < 0.001) and HER2+ tumors (HR 2.78; 95% CI, 1.27 to 6.06; P = 0.008). In ER-/HER2- tumors, the accuracy of the stem cell distance-based predictor was very high in the first two years after diagnosis. However, our stem cell distance-based predictor was not able to stratify the histologically poorly differentiated ER-/HER2- tumors into groups with significantly different 10-year survival outcome (HR 1.27; 95% CI, 0.599 to 2.7; P = 0.514). This finding was not unlike the outcomes of most validated gene signatures, which also provide only moderate prognostic information for ER-/HER2- tumors [32, 33].

### New stem cell signature derived from stem cell distance

**Fig 4:**
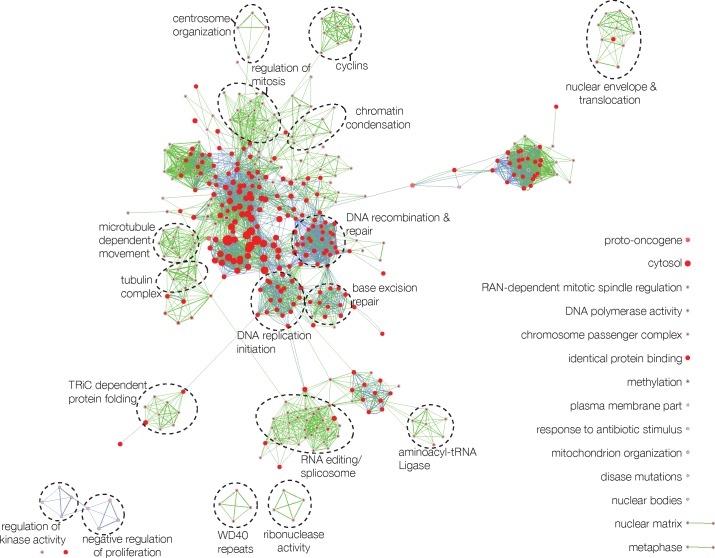
Enrichment analysis of the stem cell gene signature. Visualization of all significant gene sets enriched in our signature detected by the DAVID enrichment chart. This network shows each gene set as a node and connects these nodes based on the genes that are shared between two gene sets with the thickness of the edges being proportional to the number of genes shared between two nodes. The size of the nodes visualizes the number of genes in the signature belonging this gene set. The network of our hESC signature is shown in green edges and the center circle in a node, and for comparison we added the hESC signature by Ben-Porath et al. [9] in blue edges with outer circle on a node. Red signifies the level of significance, while grey shows nodes that are not significant in that gene set.

Finally, we generated more parsimonious prediction models by identifying top genes associated with hESC distance across all cancer subtypes (including carcinomas, sarcomas, and hematologic malignancies) in a meta-analysis approach (S2 File, described in Methods) and validated these signatures by comparing them to published stem cell signatures [9, 20]. We tested these signatures again in detail in lung adenocarcinoma and breast cancer and found identical prediction performances (Fig H in S1 File) compared to the hESC distances. The genes in our hESC signature displayed a high overlap with the signature identified by Ben-Porath et al. [9] (16%, P < 0.001). We then visualized the DAVID enrichment chart (which includes gene sets and pathways from various sources) for our hESC signature; this analysis showed that several gene sets associated with cell cycle control/division and DNA replication were significantly enriched in our signature (Fig 4). We also investigated whether the genes in our signature could be explained by the activation or inhibition of certain sets of transcription factors. To this end, we used Ingenuity transcription factor analysis; the results are displayed in Table 1, which shows the predicted activation and inhibition states of several transcription factors (TFs) as well as the number of genes in our dataset that are regulated by that TF. In all other analyzed solid tumor types (colorectal, liposarcoma and ovarian), the hMSC predictor was superior compared to the hESC distance based model. We therefore developed a hMSC signature using the same methodology and compared it again to the Ben-Porath signature. The Ben-Porath signature did not achieve concordances significantly different from random predictions in any of three remaining histologies, while our signature achieved a moderate, but statistically significant CPE of 0.55 (95% CI 0.53–0.58). We further developed a CD34+ signature for hematopoietic malignancies, which was slightly less accurate (CPE 0.54, 95% CI 0.52–0.56) than a recently proposed hematopoietic stem cell signature [20] (CPE 0.56, 95% CI 0.53–0.58) for predicting patient survival (Fig H in S1 File).

**Table 1:** Predicted activation/inhibition states of transcription factors based on our hESC gene signature. The table shows the Ingenuity analysis for prediction of top 5 activated or inhibited transcription factors (TFs). The p-value of the overlap is calculated using Fisher’s exact test and indicates the overlap between the signature genes and genes regulated by that TF.

Transcription Factor	Predicted activation state	Number of genes in our signature regulated by the TF	P-value of overlap
MYC	Activated	85	3.64E-43
TBX2	Activated	30	9.72E-32
E2F1	Activated	71	4.58E-53
FOXM1	Activated	25	2.33E-28
FOXO1	Activated	16	2.49E-06
TP53	Inhibited	109	6.47E-51
CDKN2A	Inhibited	43	4.09E-33
RB1	Inhibited	49	2.55E-36
SMARCB1	Inhibited	26	9.17E-17
KDM5B	Inhibited	20	5.81E-14

## Discussion

Here we have presented a novel computational methodology and analyses rooted in an understanding of the biologic relationship between cellular differentiation and carcinogenesis. We hypothesized that information about differentiation, which is usually provided by histologic examination of tumor cell populations, should also be contained in tumor gene expression. Just as histologic determination of levels of differentiation is oriented between the poles of stem cells and fully differentiated cells (e.g. stem cells have a high nuclear-to-cytoplasm ratio and have few [~300] mitochondria, while differentiated cells have a low nuclear-to-cytoplasm ratio and contain many [~3000] mitochondria)], we hypothesized that cancer gene expression is best analyzed by orienting it between the poles of the expression of stem cells and of fully differentiated tissue. Thus, we conjectured that the distance of a tumor sample’s gene expression from the expression of stem cells would organize tumors in a clinically coherent fashion and be predictive of patient survival. Our goal was to create a method for orienting cancers within a single framework that is (i) clinically coherent (i.e. be concordant with clinically recognized prognostic variables such as presence of nodal metastases), (ii) comprehensive (i.e. including epithelial, mesenchymal, hematopoietic malignancies), (iii) prognostic, and (iv) mechanistic (allowing analyses of the underlying biology of the disease). Most prior reports examine whether stem cell-associated ‘factors’ are found in poorly differentiated or poor prognosis cancers [34–43]. The majority of the remaining reports extend this work to investigate if a previously derived limited number of genes expressed in stem cells (a stem cell ‘signature’), if expressed in cancers, stratifies the latter by prognosis [8, 9, 44–60]. A few publications compare the similarities between cancers and stem cells based on methylation patterns [61–63] or chromatin states [64]. Only one prior publication reports examining global gene-expression between stem cells and cancers [65]. In this work, the authors provide a comparison between the gene expression of stem cells and of breast cancers of different histologies. There is no correlation with clinical data including prognosis, nor integration of diverse histologies, nor exploration of how this model illuminates the underlying biology of cancer. In contrast, when applied to multiple diverse histologies, our methodology demonstrated clinically coherent associations between tumor distance from stem cell gene expression and clinic-pathological variables well known to be associated with survival such as the degree of differentiation, tumor size, the finding of nodal metastases, and glucose uptake on ^18^F-FDG-PET (Fig 1) as well as patient survival (Fig 2). Tumors most similar in expression to stem cells were histologically more poorly differentiated, larger, more likely to be node positive, and more FDG avid on PET imaging. Consistent with this, for every histology analyzed, the tumors most similar in expression to stem cells (i.e. the most undifferentiated) were also associated with a poorer prognosis. Note that our goal was to provide a method that can be applied to all cancer types regardless of availability of data on tissue-specific stem cells. We have therefore investigated only a limited number of different stem cell datasets.

Our findings highlight the relationship between cancer and the evolutionary emergence of multicellularity. Our hESC-oriented signature shows that the most undifferentiated cancers of diverse histologies share a common pattern of gene expression. When the gene expression of the cancers most similar to stem cells of all seven histologies (lung, breast, colon, ovary, leukemia, lymphoma, and liposarcoma) were analyzed, up- or downregulation of a limited number of genes was found; in particular, we found upregulation of oncogenes (including MYC, TBX2) and down regulation of tumor suppressor genes (including p53 and RB1) (Table 1). The majority of the members of this group of transcription factors have been noted by developmental biologists to be highly evolutionarily conserved, likely due to their role in the regulation of proliferation, differentiation, and apoptosis. For example, the transcription factor Myc responds to extracellular signals by regulating cell proliferation, growth, differentiation and apoptosis; a homolog is found in choanoflagellates and is phylogenetically conserved in metazoans [37]. Similar analyses have demonstrated a high level of evolutionary conservation of the other members of the transcription factors regulating our hESC-oriented signature including p53 [40], RB [35], the CDK family [42], T box genes [39], and the Forkhead family (FOXO3) [41]. Our hESC signature therefore is consistent with a model of multiple cell divisions leading to the accumulation of mutations within resulting daughter cells causing dysfunction of multiple highly evolutionarily conserved pathways regulating growth, proliferation, differentiation, and apoptosis controlled by oncogenes and tumor suppressors genes. This leads to a cell that neither differentiates nor responds to the internal and external signals of a multicellular state and so reverts to a single cell state [38].

The wide-ranging applicability of our approach suggests that a stem cell distance-based predictor will prove useful for survival prediction across a wide variety of diverse cancer types. Our method can be applied to other cancer datasets without training, by using parameters that displayed a robust performance in most cancer types (Table M in S1 File). Furthermore, our methodology showed stable prediction when used for different cohorts. We have further shown that our methodology can be extended to produce parsimonious prediction models based on limited numbers of genes in a clean meta-analysis framework. Our hESC signature was statistically significantly similar to a signature obtained by a meta-analysis of 20 hESC transcriptome profiling studies [9] (Fig 4), demonstrating the robustness of our computational methodology and its usefulness for developing novel cancer type specific stem cell signatures. The stem cell distance derived from these signatures is promising to become a novel single prognostic feature, much as tumor size is, that can be used for a wide range of cancer histologies.

Several caveats apply to our work. Given larger sample sizes, it is likely possible to find single gene signatures with better prediction accuracies than ours. The stem cell distance, while more robust than most other tested signatures for breast cancer and lung adenocarcinoma, was not statistically significantly better than the best published predictors. However, next generation prediction models could combine thoroughly tested biologically motivated signatures such as our signature; such models could assign tumor samples a score for each cancer hallmark [66]. This might lead to robust and biologically motivated prediction models. Further work is needed to establish signatures for these other hallmarks. While our approach demonstrated promise in AML and DLBCL, the prognostic potential of the stem cell distance was less pronounced for these data sets than in the tested solid tumors (Table L in S1 File). Finally, the number of stem cells profiled on standard Affymetrix arrays is limited. Further work is needed to explore the influence of experimental conditions on gene expression and the corresponding impact on the stem cell distance of patient samples.

## Supporting information

S1 File Supplementary figures and tables. (PDF)

S2 File Supplementary tables A-D.
(XLSX)
