# Supplementary material for: Distance in cancer gene expression from stem cells predicts patient survival
Source: PLoS One. 2017 Mar 23;12(3):e0173589. doi: 10.1371/journal.pone.0173589 (PMC5363813; doi:10.1371/journal.pone.0173589)
Supplement: S1 File — (PDF) [file pone.0173589.s001.pdf]

# Distance in cancer gene expression from stem cells predicts patient survival - Supplemental Information

Markus Riester, Hua-Jun Wu, Ahmet Zehir, Mithat Gönen, Andre L. Moreira, Robert J. Downey, Franziska Michor

March 9, 2017

## Contents

**1 Datasets** **1**
1.1 Stem Cells . . . . . . . . . . 1
1.2 Lung Adenocarcinoma . . . . . . . . . . 2
1.3 Breast Cancer . . . . . . . . . . 2
1.4 Liposarcoma . . . . . . . . . . 2
1.5 Colorectal Cancer . . . . . . . . . . 2
1.6 Ovarian Cancer . . . . . . . . . . 2
1.7 Hematological Malignancies . . . . . . . . . . 2
1.8 Clinical Data . . . . . . . . . . 2
1.9 Preprocessing . . . . . . . . . . 3

**2 Parameter identification** **3**
2.1 Optimal parameters for the stem cell distance calculation . . . . . . . . . . 3

**3 Kaplan-Meier analysis** **5**

**4 Comparison to other methods** **8**
4.1 The hESC predictor is superior to many other predictors . . . . . . . . . . 8

**5 Clinicopathologic covariates and treatment response** **9**
5.1 No significant association between stem cell distance and response to adjuvant chemotherapy in adenocarcinoma of the lung . . . . . . . . . . 9

**6 New stem cell signatures derived from hESC distances** **9**

**7 Supporting Tables** **12**

**Supplemental References** **22**

## 1 Datasets

### 1.1 Stem Cells

Raw expression data of human embryonic stem cell (hESC) and human mesenchymal stem cell (hMSC) lines was acquired from the Gene Expression Omnibus (GEO[1]) (GSE7332[2]). Data on hematopoietic progenitors was also obtained from GEO (GSE9476[3], GSE30377[4]).

## 1.2 Lung Adenocarcinoma

CEL files and annotations of the adenocarcinoma samples were acquired from NCI caArray[5], accession id *jacob-00182*. The sample collection was conducted by the Director's Challenge (DC) project, a consortium of four different institutions: University of Michigan Cancer Center (UM), Moffitt Cancer Center (HLM), Memorial Sloan-Kettering Cancer Center (MSK) and the Dana-Farber Cancer Institute (DF). In our study, we divided the data into training and validation datasets as previously done by Shedden et al.[6], who used 254 samples from two cohorts (UM/HLM) as training set and two validation datasets containing 104 (MSK) and 82 (CAN/DF) samples, respectively. The 82 CAN/DF samples included 43 samples from the University Health Network (UHN) and 39 samples from the National Cancer Institute of Canada Clinical Trials Group (NCIC CTG). In addition, 28 CAN/DF adenocarcinoma samples not included in the DC study were downloaded from GEO (GSE14814[7]). The acronyms used to label these three datasets are the same as those used in the original publication[6]. Treatment information was acquired for 71 CAN/DF samples from `http://www.cs.utoronto.ca/~juris/data/JCO10/`. These 28 additional samples were not included in the CAN/DF validation set, but were only used for post-surgical treatment response analysis.

## 1.3 Breast Cancer

We used already normalized breast cancer expression data available in Bioconductor[8], comprising 1,130 samples: UPP: GSE3494[9]; MAINZ: GSE11121[10]; UNT: GSE2990, GSE6532[11]; TRANSBIG: GSE7390[12]; VDX: GSE2034[13,14]. Names of these datasets correspond to their names in Bioconductor (package names breastCancer*Dataset*). The UPP dataset was used for training, while the MAINZ, UNT, TRANSBIG, and VDX datasets were used for validation. Tumors were classified by subtype using a Gaussian mixture model[15], implemented in the Bioconductor genefu package (*scmgene* function).

## 1.4 Liposarcoma

We acquired raw data of 83 and 62 liposarcoma samples from GEO (GSE21124[16] and GSE21050[17]). Survival information for GSE21124 was obtained from the authors of the study.

## 1.5 Colorectal Cancer

Data from two datasets (226 patients from GSE14333 and 232 from GSE17538) with available disease-specific outcome information was acquired from the curatedCRCData Bioconductor package. Only probe sets also present on the Affymetrix U133A chip were used.

## 1.6 Ovarian Cancer

Data from three datasets (511 TCGA samples, 185 samples from GSE26712, and 278 samples from GSE9891) were downloaded from the curatedOvarianData package.

## 1.7 Hematological Malignancies

Raw data of 163 (GSE12417[18]) and 516 (GSE14468[19]) AML samples, 123 (GSE4475[20]) and 414 (GSE10846[21]) diffuse large B-cell lymphoma samples were obtained from GEO. Survival data for the GSE14468 AML dataset was obtained from the authors.

## 1.8 Clinical Data

A waiver of authorization to access associated clinical data was obtained from the MSKCC Institution Review Board. Demographic and clinical information for the DC validation cohort of lung adenocarcinoma patients from MSKCC (MSK cohort)[6,22] and for the MSKCC liposarcoma patients[16] was obtained from a retrospective

review of the prospectively maintained Thoracic Service and Gastric and Mixed Tumor Service databases. PET SUV values were derived from the PET scan reports. Pathologic specimens from the MSK cohort of lung adenocarcinomas were reviewed by one pathologist (A.M.) and classified as solid (poor differentiation), acinar (moderate differentiation), and papillary (well differentiation).

### 1.9 Preprocessing

We obtained $\log_2$ transformed expression estimates with the GC robust multi-array average (GCRMA) algorithm[23]. For lung adenocarcinoma samples, survival data was censored at month 60 as in[6]. For breast cancer, we censored at month 120, because of the available follow-up time and high 5-year survival rates. Other cancer types were censored at month 60 (liposarcoma, colorectal cancer and AML) or 120 (B-cell lymphoma) based on the available follow-up time.

## 2 Parameter identification

### 2.1 Optimal parameters for the stem cell distance calculation

In the main text, we show the influence of stem cell data and gene filter on the prediction accurary using Pearson Correlation distance. We tested the association of stem cell distances with outcome using alternative distance metrics and visualize the results in Supplemental Figure A. Models using Pearson Correlation distance were in most cases superior to models using Euclidean or Mutual Information distance.

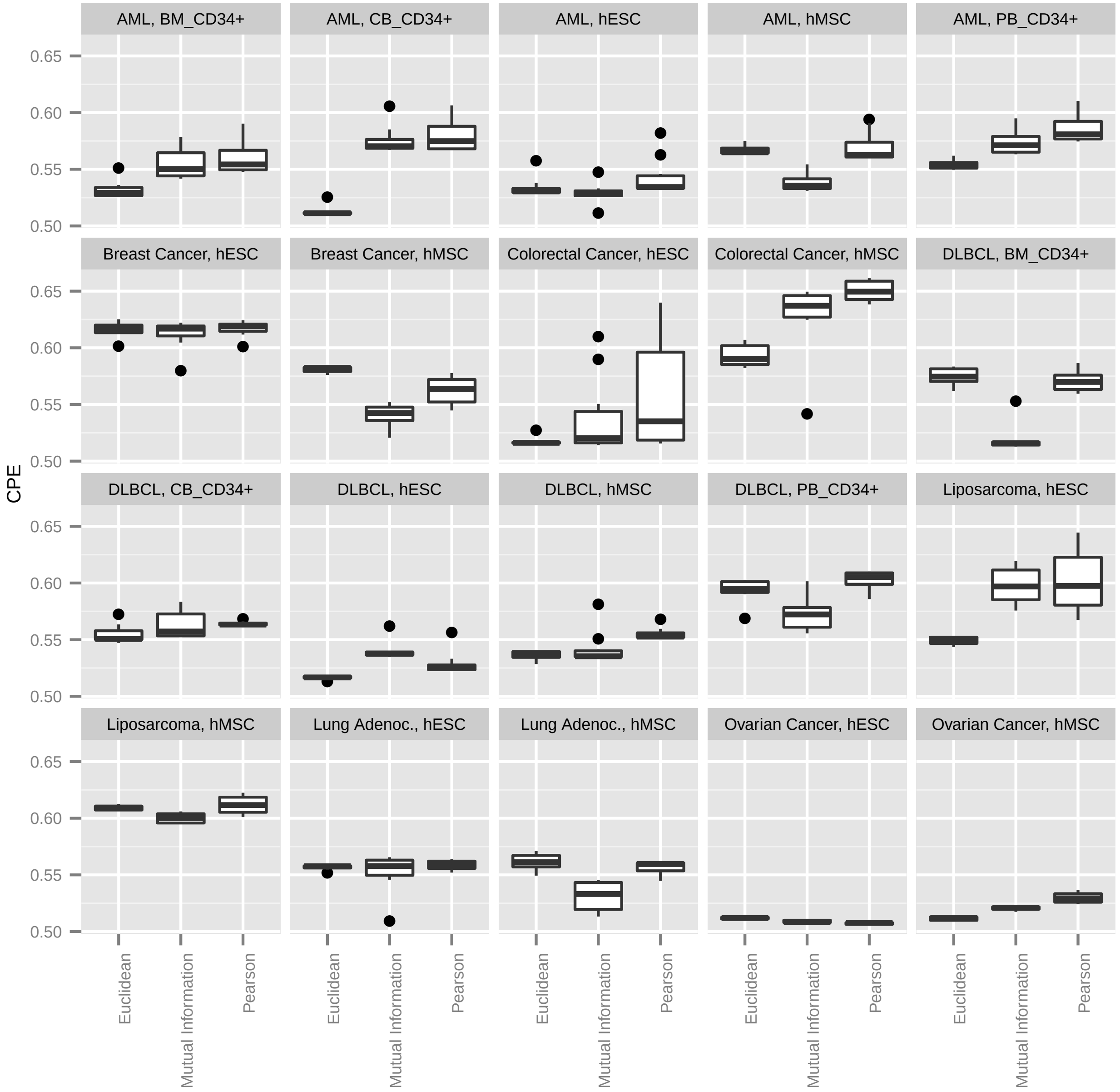


Supplementary Figure A: Influence of the distance metric on the prediction accuracy. The accuracy of a univariate Cox proportional hazards model based on the distance $d$ to stem cell was examined for three distance metrics (Pearson Correlation, Mututal Information and Euclidean) in all cancer and stem cell type combinations. The prediction accuracy is reported as the concordance probability estimate (CPE). Shown are, via box plots, the distributions of CPEs for all gene filter cutoffs. Error bars indicate the largest value with 1.5 times the third quartile and the lowest value larger than 2/3 the first quartile (the common standard for box plots). Values outside this range are visualized as dots and considered outliers.

# 3 Kaplan-Meier analysis

In addition to the Kaplan-Meier plots in Fig. 2A, we present the results of a detailed Kaplan-Meier analysis including hazard ratios (HRs) and patient numbers in all lung adenocarcinoma (Supplementary Figure B), breast cancer (Supplementary Figure C) datasets as well as all other cancer types (Supplementary Figure F). Hazard ratios compare the survival of high-risk and low-risk patients. In colorectal cancer[24], the hESC distance is predictive of survival when adjusting for stage. In Supplementary Figure F, we show Kaplan-Meier curves of stage B and C (Dukes staging), in which we stratified patients based on the hESC distance cohort median.

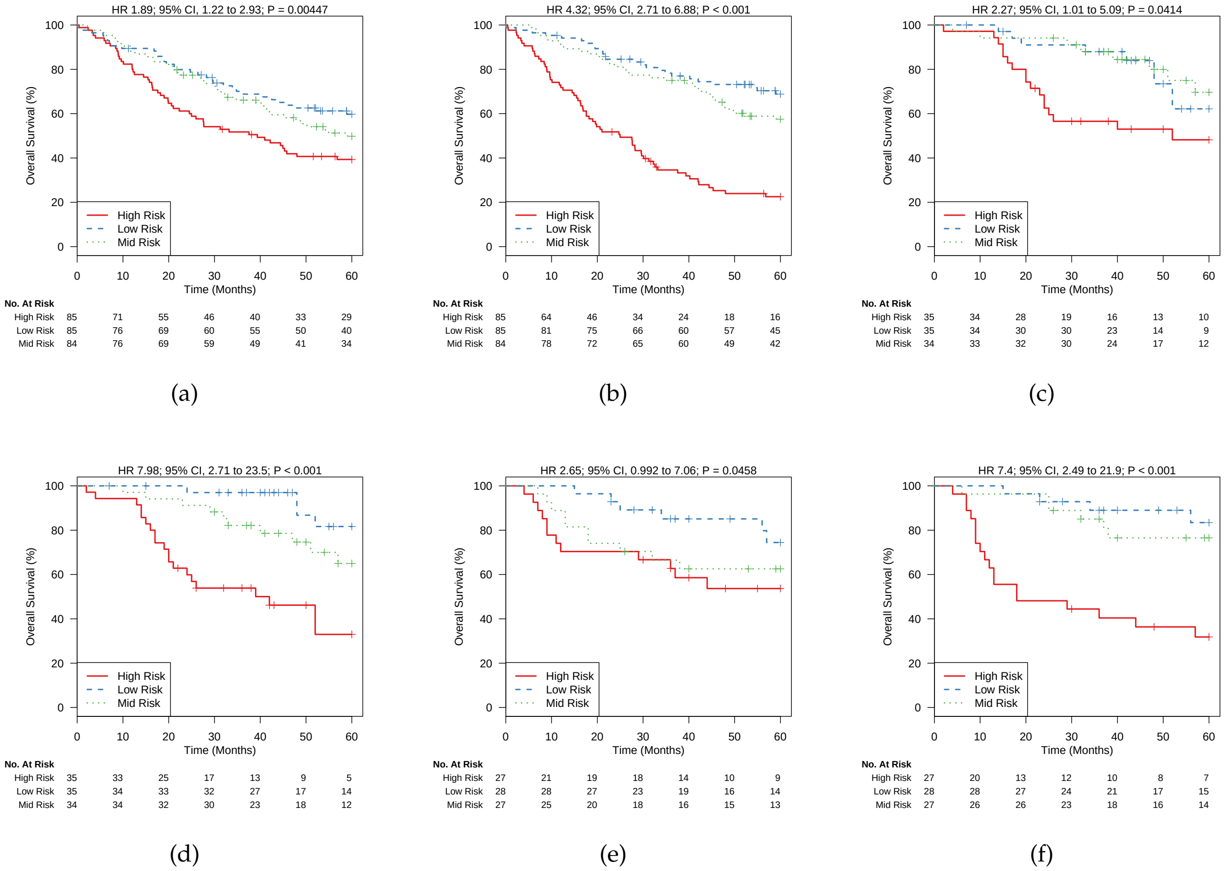


Supplementary Figure B: Kaplan-Meier curves of the Director's Challenge datasets. Risk stratification based on distance of a sample's gene expression from stem cell expression. The continuous stem cell predictor was trichotomized based on the cohort tertiles. Samples with expression distance close to stem cells smaller were classified as high-risk, others as low-risk. Hazard ratios (HR) indicate survival differences between the high- and the low-risk group. (a) Training sets UM and HLM; (b) Training sets UM and HLM with covariates Age, Gender, T and N Stage. (c) MSK; (d) MSK with covariates; (e) CAN/DF; (f) CAN/DF with covariates. In (c-f), the validation sets used a Cox model fitted in the UM/HLM tuning set.

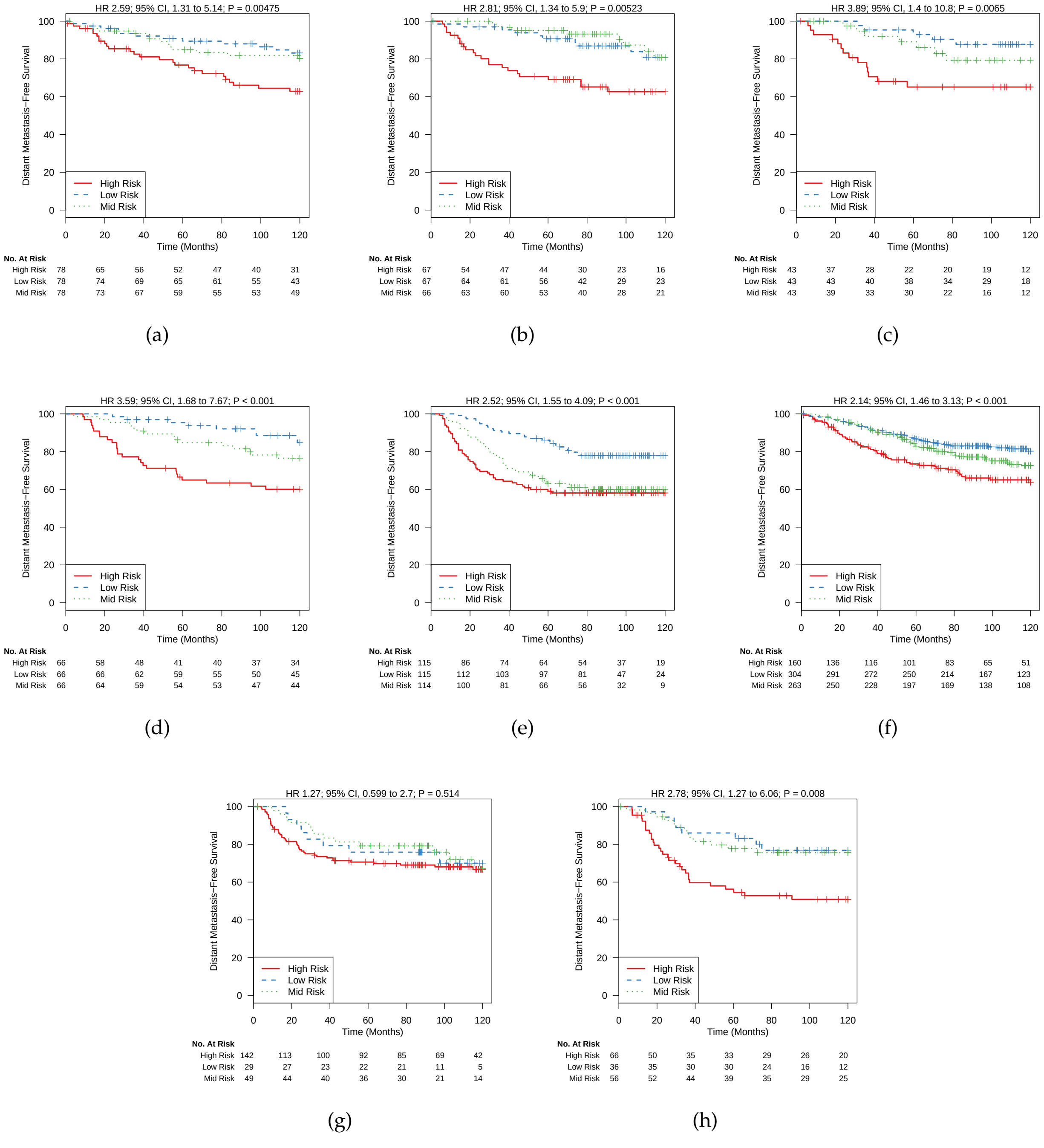


Supplementary Figure C: Application of the method using distance from stem cell expression as measure of prognosis for breast cancer. (a) Training set UPP; (b) MAINZ; (c) UNT; (d) TRANSBIG (e) VDX; (f) all ER+/HER2- from all datasets; (g) all ER-/HER2-; (h) HER2+.

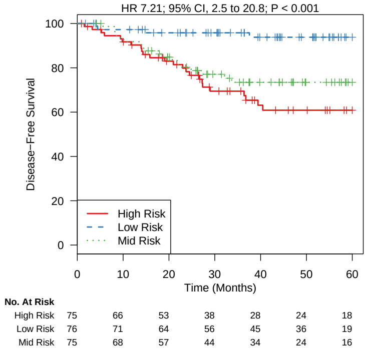


(a) Colorectal cancer (Tuning Dataset, A-C Stage)

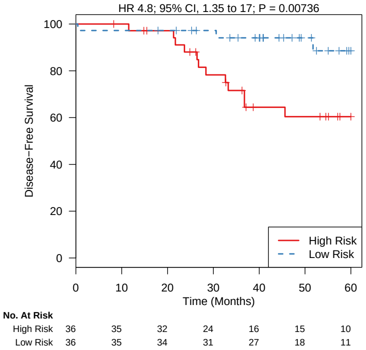


(b) Colorectal cancer (B Stage, all datasets)

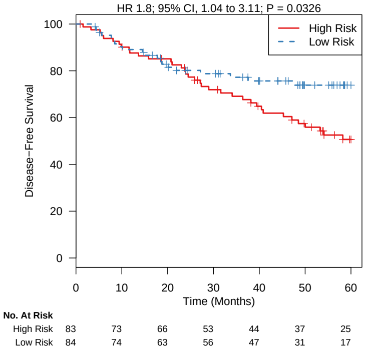


(c) Colorectal cancer (C Stage, all datasets)

Supplementary Figure D: Risk stratification based on stem cell distance in colorectal cancer. See Figure 2 in the main paper for the Kaplan-Meier plot of the validation dataset GSE17538.

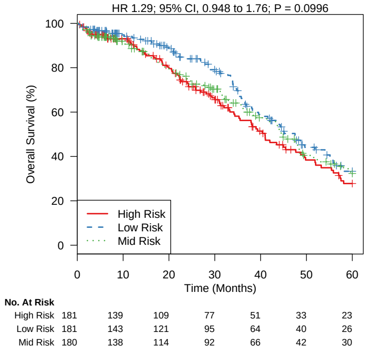


(a) Ovarian cancer (Tuning Dataset TCGA)

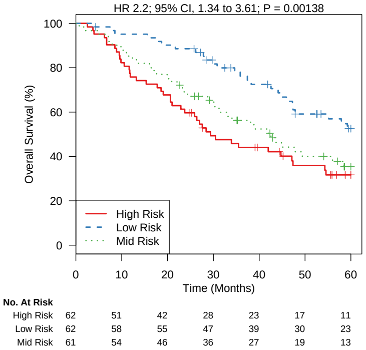


(b) Ovarian cancer (GSE26712)

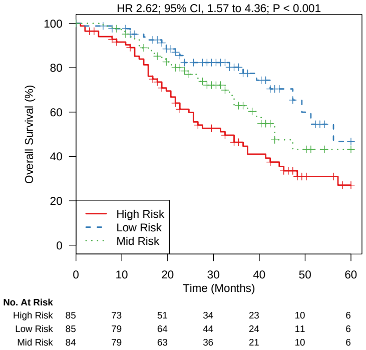


(c) Ovarian cancer (GSE9891)

Supplementary Figure E: Risk stratification based on stem cell distance in all ovarian cancer datasets.

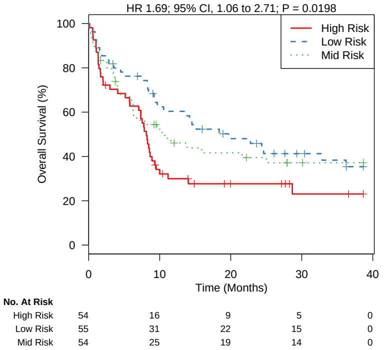


(a) AML

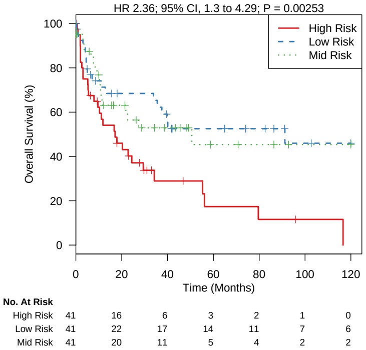


(b) DLBCL GSE4475

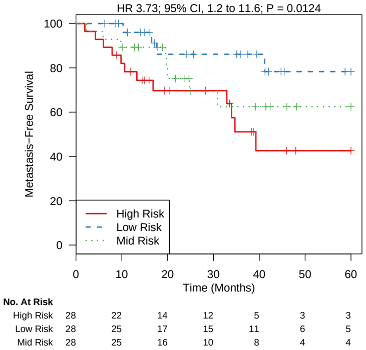


(c) Liposarcoma

Supplementary Figure F: Risk stratification in the tuning datasets of the remaining cancer types. See Figure 2 in the main paper for the Kaplan-Meier plots of the validation datasets.

# 4 Comparison to other methods

## 4.1 The hESC predictor is superior to many other predictors

We then aimed to compare our predictor to other methods. In addition to the predictors of the DC study[6], we further included other predictors[25,7,26] from papers citing the DC study into our analysis. Furthermore, we compared the predictors to a simple method based on a principal component analysis (PCA). Here, the principal components of the expression matrix were determined in the tuning data. Then, a Cox proportional hazards model was fitted using the first $n$ dimensions of the PCA scaled expression matrix. Validation expression data was scaled with the prinicipal components determined in the tuning data and the Cox model was then used to calculate risk scores. We considered the first three principal components and used the number of components with maximum concordance (CPE) in the tuning dataset, determined by 5-fold cross-validation. Only probe sets with IQR larger than the median IQR in the tuning data were considered.

This approach represented a methodology that, similarly to ours, summarizes the expression information of thousands of genes instead of using only a small number of marker genes. The PCA-based predictor was also applied to the breast cancer cohorts. The complete results of the comparison are provided in Supplementary Tables D-G for adenocarcinoma of the lung and Supplementary Tables H-I for breast cancer. Our hESC predictor was superior to all other predictors. The predictor based on the first three principal components (PCA3) displayed on average the lowest prediction concordance.

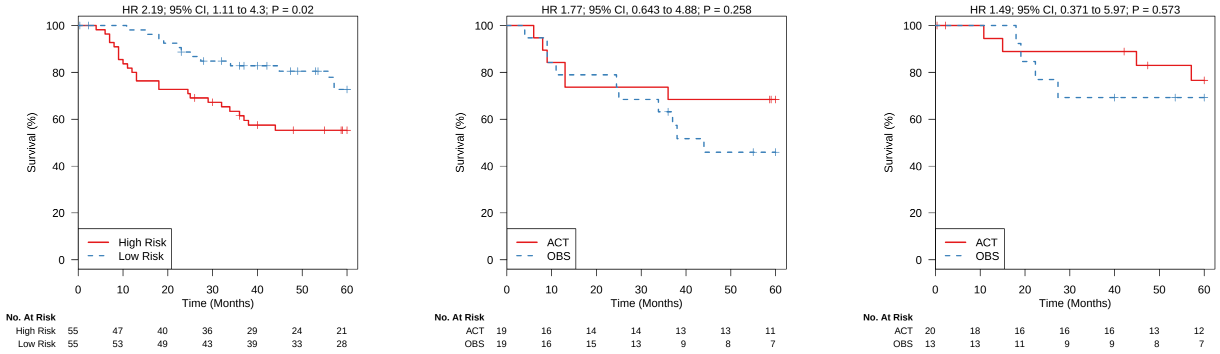


(a) (b) (c)

Supplementary Figure G: Kaplan-Meier of the SC predictor, fitted in the UM/HLM tuning data set and applied to the Zhu et al.[7] data, which consists of all low stage CAN/DF samples as well as 28 additional low stage samples. (a) hESC predictor, fitted in the UM/HLM tuning set; (b) high-risk group patients (defined with the hESC predictor) in the adjuvant chemotherapy (ACT) and the control (OBS) arms; (c) low-risk group patients in the ACT and OBS arms.

# 5 Clinicopathologic covariates and treatment response

We listed all hazard ratios of univariate models for all cancer types and cohorts and compared them with multivariate models utilizing established prognostic factors in Supplementary Table L. In AML, hESC distance lost its significance after adding establish clinicopathologic covariates. Although both univariate models were significant, hESC could not add more surival information to standard staging.

## 5.1 No significant association between stem cell distance and response to adjuvant chemotherapy in adenocarcinoma of the lung

We tested whether stem cell distance was associated with a response to adjuvant chemotherapy in low-stage lung adenocarcinoma patients (stage I and II). Zhu et al.[7] reported a gene signature for early stage non-small cell lung cancer patients and demonstrated that patients classified as high-risk based on their signature benefited significantly from adjuvant chemotherapy while patients in the low-risk group did not. We therefore tested whether the distance of a sample in gene expression from that of stem cells was associated with a response to adjuvant chemotherapy in low-stage patients (stage I and II).

A univariate Cox model with hESC distance as covariate was trained on the UM/HLM low stage patients. The Zhu et al.[7] patients, some of which were part of the CAN/DF dataset, were then stratified into low- and high-risk groups based on the median of the predicted risk scores in the dataset. Kaplan-Meier curves were then generated for the two arms (adjuvant chemotherapy versus control) in the two risk groups (Supplementary Figure G). The stratification resulted in groups with significantly different survival in the test dataset (HR 2.19; 95% CI, 1.11 to 4.3; P = 0.02). While we observed a higher 5 year survival rate in the adjuvant chemotherapy arm in the high-risk group, the difference was not significant (HR 1.77; 95% CI, 0.643 to 4.88; P = 0.258). This analysis should be repeated when larger sample sizes become available; the current sample size of 71 patients with treatment information is likely too small for further stratification by arms.

# 6 New stem cell signatures derived from hESC distances

We next sought to obtain a robust gene signature from the hESC distances. We utilized a meta-analysis approach in which we first identified the top 500 genes associated with hESC distance. In a second step, this hESC signature was then used as a prediction model utilizing the pooled regression coefficients to calculate a patient

risk score as previously described[27]. This score describes the stem cell similarity of a sample and was then evaluated for predicting outcome in a Cox model.

We compared this new hESC signature with the hESC associated genes published by Ben-Porath et al.[28,29] which achieved similar prediction concordances (Supplementary Figure H). We used the same approach for hematological malignancies and compared the results with a recent hematopoietic stem cell signature (Supplementary Figure H). Note that the hematopoietic stem cell signature used one AML validation cohort (GSE12417) for training, thus the overall concordance is biased because of the high prediction concordance in this cohort.

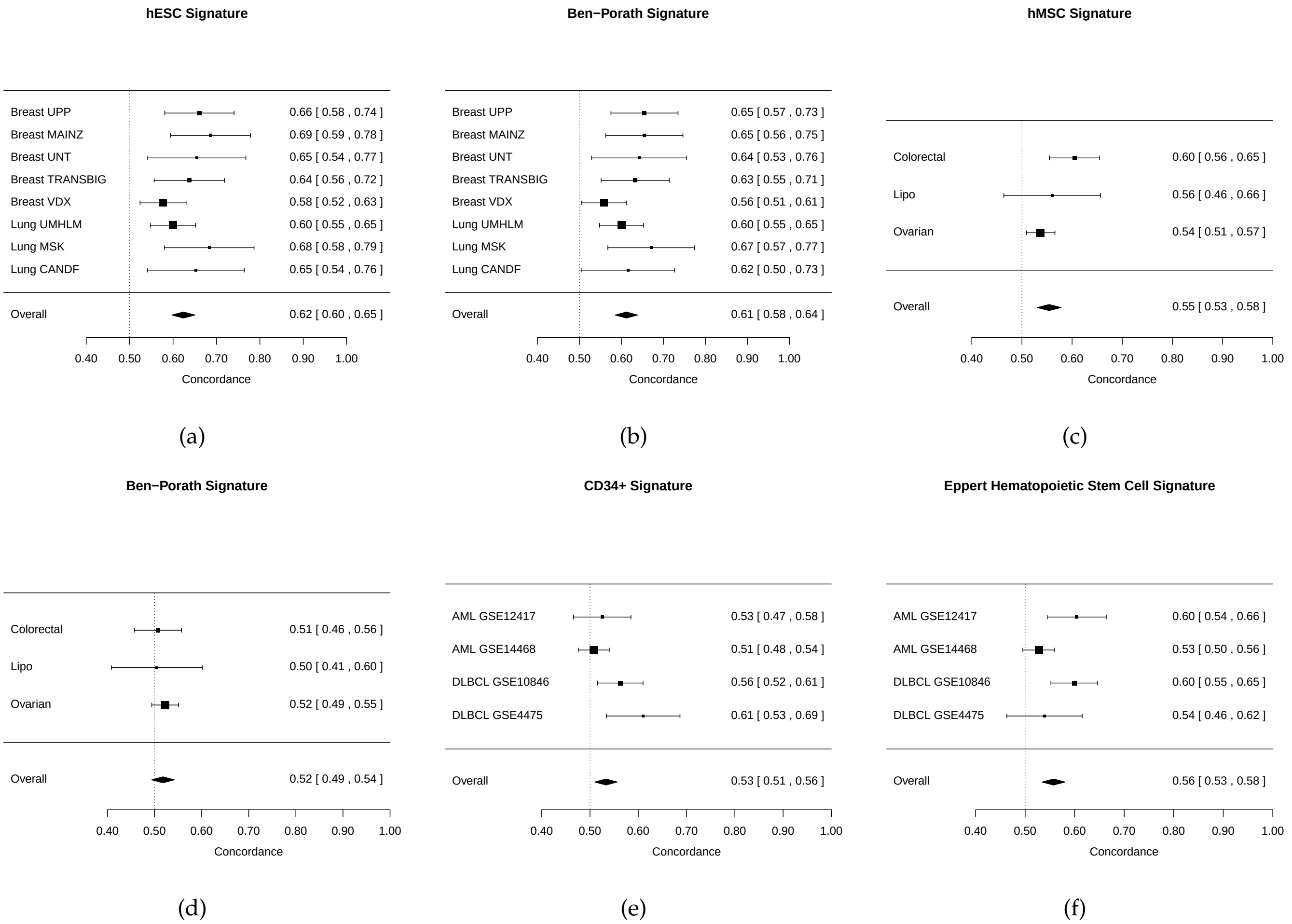


Supplementary Figure H: (a) Forest plot of our hESC signature in all breast cancer and lung adenocarcinoma cohorts, the two histologies in which the hESC distance showed better survival concordance compared to the hMSC distance. For each cohort, we built a model by using only the remaining cohorts. (b) Forest plot of the Ben-Porath signature[28] in all breast cancer and lung adenocarcinoma cohorts. The Ben-Borath model was trained using their signature genes only on the same set of training data as in (a). (c) The same approach as in (a) was utilized for a hMESC signature in all other solid tumors and again compared to the Ben-Porath model (d). The Ben-Porath model was again re-trained (i.e. the weights [pooled Cox coefficients] of each gene in the signature were re-calculated) on the same set of datasets as in (c) to ensure a fair comparison. CD34+ stem cell signature in our AML and DLBCL cohorts. (d) Comparison to a recent hematopoietic stem cell signature[4], which was trained on the AML (GSE12417) dataset.

# 7 Supporting Tables

Supplementary Table A: Datasets used in this study. Number of patients correspond to the number of patients in the dataset with available survival information.

| Cancer Type | Cohort | Accession Id | Number Patients | Number Events | Used for Training | Remarks |
|---|---|---|---|---|---|---|
| Lung Adenocarcinoma | UM/HLM[6] | jacob-00182 | 254 | 124 | Yes | |
| Lung Adenocarcinoma | MSK[6] | jacob-00182 | 104 | 34 | No | |
| Lung Adenocarcinoma | CAN/DF[6] | jacob-00182 | 82 | 28 | No | |
| Lung Adenocarcinoma | CAN/DF[7] | GSE14814 | 28 | No | treatment analysis only | |
| Breast Cancer | UPP[9] | GSE3494 | 234 | 52 | Yes | 33 HER2+; 27 ER-/HER2- |
| Breast Cancer | MAINZ[10] | GSE11121 | 200 | 41 | No | 21 HER2+; 25 ER-/HER2- |
| Breast Cancer | UNT[11] | GSE6532 | 129 | 26 | No | 19 HER2+; 22 ER-/HER2- |
| Breast Cancer | TRANSBIG[12] | GSE7390 | 198 | 50 | No | 28 HER2+; 45 ER-/HER2- |
| Breast Cancer | VDX[13,14] | GSE2034 | 344 | 118 | No | 57 HER2+; 101 ER-/HER2- |
| Liposarcoma | [16] | GS21124 | 84 | 40 | Yes | |
| Liposarcoma | [17] | GSE21050 | 62 | 16 | No | |
| Colorectal Cancer | [24] | GSE14333 | 226 | 50 | Yes | |
| Colorectal Cancer | [30] | GSE17538 | 232 | 93 | No | |
| Ovarian Cancer | [31] | | 511 | 290 | Yes | |
| Ovarian Cancer | [32] | GSE26712 | 185 | 129 | No | |
| Ovarian Cancer | [33] | GSE9891 | 278 | 113 | No | |
| AML | [18] | GSE12417 | 163 | 103 | Yes | |
| AML | [34] | GSE14468 | 516 | 342 | No | |
| Diffuse Large B-Cell Lymphoma | [20] | GSE4475 | 123 | 66 | Yes | |
| Diffuse Large B-Cell Lymphoma | [21] | GSE10846 | 414 | 165 | No | |

Supplementary Table B: Association of demographic, clinical and pathologic covariates with the distance of a sample from stem cell expression patterns and their distribution among risk groups, based on cohort tertiles, in adenocarcinoma of the lung. Patients numbers do not sum up to the total number of patients in the DC study[6], because not all covariates were available for all patients. Fisher's exact test was used to estimate non-random distributions of the corresponding categorical covariates across the stem cell derived risk groups. For continuous covariates, the Pearson Correlation coefficient is given.

| Covariate | High-Risk Patients | Mid-Risk Patients | Low-Risk Patients | p-value | rho | Test |
|---|---|---|---|---|---|---|
| Histology (well diff.) | 4 (6.7%) | 18 (30%) | 38 (63.3%) | $<$ 2.22e-16 | | Fisher's exact |
| Histology (poor diff.) | 93 (56%) | 46 (27.7%) | 27 (16.3%) | $<$ 2.22e-16 | | Fisher's exact |
| Histology (moderate diff.) | 51 (24.4%) | 82 (39.2%) | 76 (36.4%) | $<$ 2.22e-16 | | Fisher's exact |
| Stage (T1) | 31 (20.7%) | 57 (38%) | 62 (41.3%) | 0.00013 | | Fisher's exact |
| Stage (T2) | 103 (41%) | 75 (29.9%) | 73 (29.1%) | 0.00013 | | Fisher's exact |
| Stage (T3/4) | 17 (43.6%) | 15 (38.5%) | 7 (17.9%) | 0.00013 | | Fisher's exact |
| Gender (female) | 66 (28.3%) | 68 (29.2%) | 99 (42.5%) | 0.00016 | | Fisher's exact |
| Gender (male) | 90 (38.3%) | 88 (37.4%) | 57 (24.3%) | 0.00016 | | Fisher's exact |
| SUV | | | | 0.00083 | -0.615 | Pearson Correlation |
| Stage (N0) | 97 (29.6%) | 106 (32.3%) | 125 (38.1%) | 0.00172 | | Fisher's exact |
| Stage (N1/2) | 59 (42.1%) | 50 (35.7%) | 31 (22.1%) | 0.00172 | | Fisher's exact |
| Smoking (never) | 12 (25%) | 10 (20.8%) | 26 (54.2%) | 0.00321 | | Fisher's exact |
| Smoking (currently) | 17 (53.1%) | 10 (31.2%) | 5 (15.6%) | 0.00321 | | Fisher's exact |
| Smoking (quit) | 89 (33.3%) | 93 (34.8%) | 85 (31.8%) | 0.00321 | | Fisher's exact |
| Tumor size | | | | 0.09262 | -0.214 | Pearson Correlation |
| EGFR | 2 (13.3%) | 6 (40%) | 7 (46.7%) | 0.1626 | | Fisher's exact |
| KRAS | 2 (18.2%) | 5 (45.5%) | 4 (36.4%) | 0.23886 | | Fisher's exact |
| TP53 | 8 (30.8%) | 5 (19.2%) | 13 (50%) | 0.83053 | | Fisher's exact |
| Age (<65) | 77 (32.4%) | 80 (33.6%) | 81 (34%) | 0.91884 | | Fisher's exact |
| Age (>=65) | 79 (34.3%) | 76 (33%) | 75 (32.6%) | 0.91884 | | Fisher's exact |

Supplementary Table C: Association of demographic, clinical and pathologic covariates with the distance of a sample from stem cell expression patterns and their distribution among risk groups in breast cancer. Patients numbers do not sum up to the total number of patients in all breast cancer datasets, because not all covariates were available for all patients. Because of the large sample sizes, the $\chi^2$ test is used instead of Fisher's exact test.

| Covariate | High-Risk Patients | Mid-Risk Patients | Low-Risk Patients | p-value | rho | Test |
|---|---|---|---|---|---|---|
| Histology (well diff.) | 30 (18.8%) | 60 (37.5%) | 70 (43.8%) | 0 | | Chi Square |
| Histology (poor diff.) | 157 (45.6%) | 100 (29.1%) | 87 (25.3%) | 0 | | Chi Square |
| Histology (moderate diff.) | 131 (30.3%) | 160 (37%) | 141 (32.6%) | 0 | | Chi Square |
| Tumor size | | | | 4e-05 | -0.148 | Pearson Correlation |
| Age (<54) | 168 (31%) | 194 (35.8%) | 180 (33.2%) | 0.29904 | | Chi Square |
| Age (>=54) | 170 (33.7%) | 158 (31.3%) | 177 (35%) | 0.29904 | | Chi Square |

Supplementary Table D: Hazard ratios for lung adenocarcinoma samples from the Memorial-Sloan Kettering Cancer Center (MSK). In this table, we report the hazard ratios together with 95% confidence intervals of the quantile normalized classifier risk scores. A hazard ratio significantly larger than 1.0 indicates that patients with high risk score had poor outcome. Note that a high-risk score corresponds to low stem cell distance; the HR of stem cell distance would be thus the inverse of the reported HR (i.e., significantly smaller than 1.0). We further list three different prediction error estimates: the concordance probability estimate, the C-statistic (another concordance estimator) and the Brier Score. See *Materials and Methods* for explanations. Classifier hESC is our stem-cell distance based predictor. PCA3 uses the first 3 prinicipal components of the expression matrix. The rest are published classifiers, in which the lack of the suffix *_r* indicates that these are published, non-reproduced results.

| Classifier | HR (95% CI) | p-value | CPE | C-statistic | Reference |
|---|---|---|---|---|---|
| hESC | 1.56 (0.94-2.61) | 0.088 | 0.576 | 0.5915 | |
| PCA3 | 1.79 (1.03-3.12) | 0.038 | 0.599 | 0.598 | |
| A | 1.83 (1.24-2.7) | 0.002 | 0.627 | | [6] |
| C | 1.74 (0.87-3.47) | 0.111 | 0.561 | | [6] |
| E | 1.44 (0.71-2.89) | 0.301 | 0.542 | | [6] |
| F | 2.65 (1.32-5.33) | 0.005 | 0.611 | | [6] |
| G | 3.19 (1.5-6.78) | 0.002 | 0.632 | | [6] |
| H | 2.71 (1.36-5.42) | 0.004 | 0.634 | | [6] |
| J | 1.1 (0.73-1.68) | 0.64 | 0.52 | | [6] |
| K | 1.41 (0.94-2.11) | 0.09 | 0.58 | | [6] |
| L | 1.23 (0.84-1.8) | 0.28 | 0.55 | | [6] |
| M | 1.51 (0.92-2.47) | 0.09 | 0.59 | | [6] |
| N | 1.56 (1.09-2.25) | 0.01 | 0.61 | | [6] |
| E_r | 1.75 (0.91-3.36) | 0.092 | 0.586 | 0.586 | [6] |
| J_r | 1.23 (0.85-1.8) | 0.275 | 0.545 | 0.5849 | [6] |
| K_r | 0.96 (0.8-1.13) | 0.596 | 0.52 | 0.4919 | [6] |
| N_r | 1.67 (1.11-2.52) | 0.014 | 0.617 | 0.6375 | [6] |
| Guo_r | 0.99 (0.98-1) | 0.05 | 0.509 | 0.5714 | [25] |
| Zhu_r | 1.14 (0.8-1.62) | 0.475 | 0.528 | 0.5615 | [7] |
| Navab_r | 1.6 (1.06-2.41) | 0.025 | 0.598 | 0.6294 | [26] |

Supplementary Table E: Hazard ratios for lung adenocarcinoma samples from the Memorial-Sloan Kettering Cancer Center (MSK). As in Supplementary Table D, but with age, stage and gender added to the listed predictors in a multivariate model.

| Classifier | HR (95% CI) | p-value | CPE | C-statistic | Reference |
|---|---|---|---|---|---|
| hESC | 2.88 (1.78-4.66) | < 0.001 | 0.688 | 0.7096 | |
| PCA3 | 3.9 (2.14-7.12) | < 0.001 | 0.709 | 0.7103 | |
| A | 2.52 (1.56-4.06) | 0 | 0.671 | | [6] |
| B | 4.05 (2.18-7.52) | 0 | 0.706 | | [6] |
| I | 3.21 (1.82-5.67) | 0 | 0.67 | | [6] |
| J | 3.14 (1.71-5.78) | 0 | 0.67 | | [6] |
| K | 2.51 (1.41-4.45) | 0 | 0.66 | | [6] |
| L | 1.8 (1.16-2.77) | 0.01 | 0.61 | | [6] |
| M | 3.9 (2.13-7.15) | 0 | 0.71 | | [6] |
| N | 2.59 (1.61-4.19) | 0 | 0.68 | | [6] |
| I_r | 2.6 (1.67-4.06) | < 0.001 | 0.66 | 0.6978 | [6] |
| I_r ridge | 2.67 (1.69-4.22) | < 0.001 | 0.669 | 0.6987 | [6] |
| J_r | 2.95 (1.8-4.84) | < 0.001 | 0.68 | 0.701 | [6] |
| K_r | 2.83 (1.66-4.85) | < 0.001 | 0.677 | 0.6716 | [6] |
| N_r | 2.28 (1.43-3.65) | 0.001 | 0.637 | 0.6959 | [6] |
| Guo_r | 2.42 (1.53-3.84) | < 0.001 | 0.663 | 0.6862 | [25] |
| Zhu_r | 2.67 (1.71-4.18) | < 0.001 | 0.675 | 0.7028 | [7] |
| Navab_r | 2.88 (1.8-4.62) | < 0.001 | 0.687 | 0.7214 | [26] |

Supplementary Table F: Hazard ratios for lung adenocarcinoma samples from the CAN/DF cohort. See Supplementary Table D for details.

| Classifier | HR (95% CI) | p-value | CPE | C-statistic | Reference |
|---|---|---|---|---|---|
| hESC | 2.31 (1.38-3.86) | 0.001 | 0.652 | 0.6343 | |
| PCA3 | 1.51 (0.86-2.66) | 0.156 | 0.577 | 0.5894 | |
| A | 1.76 (1.2-2.6) | 0.003 | 0.623 | | 6 |
| C | 1.07 (0.45-2.56) | 0.878 | 0.506 | | 6 |
| E | 0.53 (0.18-1.56) | 0.239 | 0.553 | | 6 |
| F | 0.98 (0.46-2.08) | 0.947 | 0.503 | | 6 |
| G | 1.13 (0.52-2.46) | 0.751 | 0.515 | | 6 |
| H | 2.14 (1.08-4.23) | 0.025 | 0.621 | | 6 |
| J | 2.12 (1.23-3.65) | 0.01 | 0.64 | | 6 |
| K | 2.3 (1.42-3.74) | 0 | 0.66 | | 6 |
| L | 1.48 (0.83-2.62) | 0.18 | 0.57 | | 6 |
| M | 0.93 (0.57-1.54) | 0.78 | 0.52 | | 6 |
| N | 0.76 (0.55-1.06) | 0.1 | 0.58 | | 6 |
| E_r | 0.97 (0.5-1.88) | 0.925 | 0.505 | 0.5206 | 6 |
| J_r | 1.76 (1.03-3) | 0.038 | 0.615 | 0.6047 | 6 |
| K_r | 1.04 (0.75-1.46) | 0.797 | 0.512 | 0.5263 | 6 |
| N_r | 0.98 (0.82-1.17) | 0.853 | 0.507 | 0.4914 | 6 |
| Guo_r | 1 (1-1.01) | 0.574 | 0.517 | 0.5842 | 25 |
| Zhu_r | 2.5 (1.73-3.63) | < 0.001 | 0.677 | 0.6601 | 7 |
| Navab_r | 1.93 (1.33-2.79) | < 0.001 | 0.634 | 0.6672 | 26 |

Supplementary Table G: Hazard ratios for lung adenocarcinoma samples from the CAN/DF cohort. As in Supplementary Table F, but with age, stage and gender added to the listed predictors in a multivariate model.

| Classifier | HR (95% CI) | p-value | CPE | C-statistic | Reference |
|---|---|---|---|---|---|
| hESC | 4.36 (2.36-8.03) | < 0.001 | 0.723 | 0.7416 | |
| PCA3 | 3.82 (1.99-7.34) | < 0.001 | 0.718 | 0.7088 | |
| A | 6.68 (3.3-13.52) | 0 | 0.762 | | 6 |
| B | 3.11 (1.7-5.67) | 0 | 0.69 | | 6 |
| I | 3.85 (2.02-7.53) | 0 | 0.7 | | 6 |
| J | 4 (2.4-6.68) | 0 | 0.74 | | 6 |
| K | 3.64 (2.16-6.14) | 0 | 0.72 | | 6 |
| L | 3.37 (1.73-6.58) | 0 | 0.71 | | 6 |
| M | 2.21 (1.38-3.57) | 0 | 0.66 | | 6 |
| N | 1.16 (0.75-1.79) | 0.5 | 0.54 | | 6 |
| I_r | 3.58 (2.03-6.32) | < 0.001 | 0.709 | 0.7372 | 6 |
| I_r ridge | 2.86 (1.76-4.64) | < 0.001 | 0.709 | 0.7246 | 6 |
| J_r | 3.53 (2.05-6.08) | < 0.001 | 0.723 | 0.7444 | 6 |
| K_r | 4.49 (2.58-7.82) | < 0.001 | 0.733 | 0.7763 | 6 |
| N_r | 2.45 (1.49-4.03) | < 0.001 | 0.679 | 0.6711 | 6 |
| Guo_r | 3.2 (1.82-5.62) | < 0.001 | 0.699 | 0.7079 | 25 |
| Zhu_r | 4.16 (2.5-6.9) | < 0.001 | 0.729 | 0.7693 | 7 |
| Navab_r | 3 (1.83-4.9) | < 0.001 | 0.704 | 0.7087 | 26 |

Supplementary Table H: Hazard ratios for the breast cancer predictors in all four validation datasets.

| Dataset | Classifier | HR (95% CI) | p-value | CPE | C-statistic | Reference |
|---|---|---|---|---|---|---|
| MAINZ | hESC | 2.37 (1.47-3.82) | < 0.001 | 0.648 | 0.6422 | |
| MAINZ | PCA3 | 2.46 (1.47-4.11) | 0.001 | 0.648 | 0.6421 | |
| MAINZ | AURKA | 2.4 (1.58-3.63) | < 0.001 | 0.663 | 0.6803 | [35] |
| MAINZ | GENE70 | 1.92 (1.41-2.63) | < 0.001 | 0.648 | 0.6532 | [36] |
| MAINZ | GGI | 2.48 (1.6-3.83) | < 0.001 | 0.672 | 0.6514 | [37] |
| UNT | hESC | 1.62 (1.14-2.31) | 0.007 | 0.607 | 0.6656 | |
| UNT | PCA3 | 1.83 (1.07-3.13) | 0.028 | 0.61 | 0.6275 | |
| UNT | AURKA | 1.69 (1.09-2.63) | 0.019 | 0.596 | 0.6318 | [35] |
| UNT | GENE70 | 1.34 (0.82-2.18) | 0.238 | 0.557 | 0.6112 | [36] |
| UNT | GGI | 1.69 (1.01-2.82) | 0.046 | 0.594 | 0.6365 | [37] |
| TRANSBIG | hESC | 1.73 (1.2-2.49) | 0.003 | 0.608 | 0.6489 | |
| TRANSBIG | PCA3 | 1.56 (1.05-2.34) | 0.029 | 0.584 | 0.6023 | |
| TRANSBIG | AURKA | 1.7 (1.12-2.58) | 0.013 | 0.598 | 0.6036 | [35] |
| TRANSBIG | GENE70 | 1.6 (1.04-2.46) | 0.032 | 0.58 | 0.6145 | [36] |
| TRANSBIG | GGI | 2.16 (1.36-3.43) | 0.001 | 0.628 | 0.6441 | [37] |
| VDX | hESC | 1.57 (1.22-2.01) | < 0.001 | 0.585 | 0.6142 | |
| VDX | PCA3 | 1.33 (1.01-1.75) | 0.04 | 0.554 | 0.5603 | |
| VDX | AURKA | 1.62 (1.25-2.09) | < 0.001 | 0.597 | 0.6059 | [35] |
| VDX | GENE70 | 1.27 (0.97-1.67) | 0.083 | 0.544 | 0.5698 | [36] |
| VDX | GGI | 1.42 (1.06-1.9) | 0.02 | 0.562 | 0.5767 | [37] |

Supplementary Table I: Hazard ratios for the breast cancer predictors with clinical covariates (grade and tumor size). Tumor size was unavailable in the VDX validation dataset.

| Dataset | Classifier | HR (95% CI) | p-value | CPE | C-statistic | Reference |
|---|---|---|---|---|---|---|
| MAINZ | Grade+Size+hESC | 1.82 (1.35-2.47) | < 0.001 | 0.637 | 0.6735 | |
| MAINZ | Grade+Size+AURKA | 1.76 (1.32-2.35) | < 0.001 | 0.633 | 0.6776 | [35] |
| MAINZ | Grade+Size+PCA3 | 1.86 (1.33-2.6) | < 0.001 | 0.629 | 0.6675 | |
| MAINZ | Grade+Size+GENE70 | 1.65 (1.26-2.17) | < 0.001 | 0.622 | 0.6706 | [36] |
| MAINZ | Grade+Size+GGI | 2 (1.43-2.81) | < 0.001 | 0.646 | 0.6698 | [37] |
| MAINZ | Grade+Size | 1.49 (1.16-1.92) | 0.002 | 0.608 | 0.6538 | |
| MAINZ | Grade | 3.3 (1.59-6.86) | 0.001 | 0.609 | 0.6087 | |
| UNT | Grade+Size+hESC | 2.28 (1.3-4.01) | 0.004 | 0.656 | 0.6889 | |
| UNT | Grade+Size+AURKA | 1.83 (1.09-3.08) | 0.022 | 0.621 | 0.6524 | [35] |
| UNT | Grade+Size+PCA3 | 2.1 (1.23-3.61) | 0.007 | 0.65 | 0.6823 | |
| UNT | Grade+Size+GENE70 | 1.86 (1.06-3.26) | 0.031 | 0.614 | 0.6519 | [36] |
| UNT | Grade+Size+GGI | 2 (1.15-3.46) | 0.014 | 0.628 | 0.6815 | [37] |
| UNT | Grade+Size | 1.64 (1.02-2.65) | 0.043 | 0.611 | 0.6356 | |
| UNT | Grade | 1.5 (0.64-3.51) | 0.355 | 0.553 | 0.5518 | |
| TRANSBIG | Grade+Size+hESC | 2.05 (1.4-2.98) | < 0.001 | 0.633 | 0.6699 | |
| TRANSBIG | Grade+Size+AURKA | 1.94 (1.32-2.86) | 0.001 | 0.622 | 0.6491 | [35] |
| TRANSBIG | Grade+Size+PCA3 | 1.98 (1.29-3.04) | 0.002 | 0.618 | 0.6368 | |
| TRANSBIG | Grade+Size+GENE70 | 1.79 (1.27-2.54) | 0.001 | 0.617 | 0.6497 | [36] |
| TRANSBIG | Grade+Size+GGI | 2.29 (1.5-3.51) | < 0.001 | 0.642 | 0.6756 | [37] |
| TRANSBIG | Grade+Size | 1.85 (1.25-2.72) | 0.002 | 0.613 | 0.6344 | |
| TRANSBIG | Grade | 1.57 (1.02-2.4) | 0.038 | 0.582 | 0.5823 | |
| VDX | Grade | 3.15 (1.36-7.31) | 0.008 | 0.586 | 0.5726 | |

Supplementary Table J: Hazard ratios for the ovarian cancer predictors (hESC and the TCGA gene signature) in all two validation datasets.

| Dataset | Classifier | HR (95% CI) | p-value | CPE | C-statistic | Reference |
|---|---|---|---|---|---|---|
| Bonome | hESC | 1.42 (1.11-1.82) | 0.005 | 0.567 | 0.5856 | |
| Bonome | PCA3 | 1.34 (1.07-1.67) | 0.01 | 0.566 | 0.5662 | |
| Bonome | TCGA | 1.62 (1.25-2.1) | < 0.001 | 0.59 | 0.5957 | [31] |
| Tothill | hESC | 1.56 (1.21-2) | < 0.001 | 0.589 | 0.5811 | |
| Tothill | PCA3 | 1.54 (1.13-2.09) | 0.006 | 0.575 | 0.5689 | |
| Tothill | TCGA | 1.92 (1.44-2.55) | < 0.001 | 0.619 | 0.6172 | [31] |

Supplementary Table K: Hazard ratios for the ovarian cancer predictors with clinical covariates (debulking status, age and tumor stage).

| Dataset | Classifier | HR (95% CI) | p-value | CPE | C-statistic | Reference |
|---|---|---|---|---|---|---|
| Bonome | hESC | 1.66 (1.33-2.06) | < 0.001 | 0.61 | 0.6332 | |
| Bonome | PCA | 1.62 (1.31-1.99) | < 0.001 | 0.608 | 0.6349 | |
| Bonome | TCGA | 1.7 (1.39-2.09) | < 0.001 | 0.609 | 0.6547 | [31] |
| Tothill | hESC | 2.14 (1.61-2.86) | < 0.001 | 0.652 | 0.6166 | |
| Tothill | PCA | 2.17 (1.62-2.91) | < 0.001 | 0.65 | 0.6249 | |
| Tothill | TCGA | 2.52 (1.87-3.38) | < 0.001 | 0.675 | 0.6656 | [31] |

Supplementary Table L: Hazard ratios of the hESC predictor models in all datasets used in this study, compared to models using available established clinicopathologic and demographic predictors. The hESC correlation distance was scaled with the factor 10 in this analysis to achieve meaningful hazard ratios, with a hESC value of 10 corresponding to perfect hESC similarity. ECOG PS: Eastern Cooperative Oncology Group Performance Status; FAB: French-American-British Classification; FLT3 ITD: Internal tandem duplications of the *FLT3* gene; NPM1: mutation of the *NPM1* gene; LDH: Lactate dehydrogenase level; PB: peripherial blood.

| Cohort | Characteristic | N | Events | HR | P | Concordance |
|---|---|---|---|---|---|---|
| Lung UM/HLM | hESC | 254 | 124 | 1.27 (1.06-1.52) | 0.01 | 0.58 (+-0.03) |
| Lung UM/HLM | | 254 | 124 | | < 0.001 | 0.7 (+-0.03) |
| | hESC | | | 1.29 (1.03-1.61) | 0.029 | |
| | Stage | | | 1.53 (1.24-1.89) | < 0.001 | |
| | Node | | | 2.9 (2.01-4.19) | < 0.001 | |
| | Age | | | 1.03 (1.01-1.05) | 0.005 | |
| | Grade Poorly Diff. | | | 0.98 (0.65-1.49) | 0.928 | |
| | Grade Well Diff. | | | 1.15 (0.62-2.12) | 0.665 | |
| Lung MSK | hESC | 104 | 34 | 1.32 (0.96-1.8) | 0.088 | 0.64 (+-0.05) |
| Lung MSK | | 100 | 33 | | < 0.001 | 0.81 (+-0.05) |
| | hESC | | | 1.19 (0.82-1.72) | 0.371 | |
| | Stage | | | 0.98 (0.44-2.16) | 0.952 | |
| | Node | | | 4.12 (1.77-9.58) | 0.001 | |
| | Age | | | 1 (0.96-1.03) | 0.806 | |
| | Grade Poorly Diff. | | | 4.24 (1.6-11.23) | 0.004 | |
| | Grade Well Diff. | | | 3.1 (0.71-13.57) | 0.133 | |
| Lung CAN/DF | hESC | 82 | 28 | 1.94 (1.29-2.93) | 0.001 | 0.65 (+-0.06) |
| Lung CAN/DF | | 81 | 28 | | < 0.001 | 0.79 (+-0.06) |
| | hESC | | | 2.21 (1.4-3.5) | < 0.001 | |
| | Stage | | | 0.81 (0.33-1.98) | 0.646 | |
| | Node | | | 1.84 (0.82-4.16) | 0.141 | |
| | Age | | | 1.13 (1.06-1.19) | < 0.001 | |
| | Grade Poorly Diff. | | | 0.87 (0.34-2.21) | 0.764 | |
| | Grade Well Diff. | | | 0.87 (0.25-3.01) | 0.825 | |
| Lung UM/HLM | hESC | 254 | 124 | 1.27 (1.06-1.52) | 0.01 | 0.58 (+-0.03) |
| Lung UM/HLM | | 254 | 124 | | 0.072 | 0.57 (+-0.03) |
| | hESC | | | 1.29 (1.05-1.6) | 0.017 | |
| | Grade Poorly Diff. | | | 0.87 (0.57-1.32) | 0.505 | |
| | Grade Well Diff. | | | 0.89 (0.48-1.63) | 0.703 | |
| Lung MSK | hESC | 104 | 34 | 1.32 (0.96-1.8) | 0.088 | 0.64 (+-0.05) |
| Lung MSK | | 100 | 33 | | 0.003 | 0.73 (+-0.05) |
| | hESC | | | 1.12 (0.79-1.58) | 0.523 | |
| | Grade Poorly Diff. | | | 4.41 (1.75-11.08) | 0.002 | |
| | Grade Well Diff. | | | 1.66 (0.41-6.71) | 0.48 | |
| Lung CAN/DF | hESC | 82 | 28 | 1.94 (1.29-2.93) | 0.001 | 0.65 (+-0.06) |
| Lung CAN/DF | | 81 | 28 | | 0.016 | 0.65 (+-0.06) |
| | hESC | | | 2.05 (1.3-3.21) | 0.002 | |
| | Grade Poorly Diff. | | | 0.74 (0.3-1.82) | 0.511 | |
| | Grade Well Diff. | | | 0.64 (0.19-2.1) | 0.461 | |
| Breast UPP | hESC | 234 | 52 | 2.02 (1.36-3) | < 0.001 | 0.65 (+-0.04) |
| Breast UPP | | 232 | 51 | | < 0.001 | 0.72 (+-0.04) |
| | hESC | | | 1.79 (1.07-2.97) | 0.025 | |
| | Grade | | | 1.4 (0.88-2.22) | 0.158 | |
| | Tumor Size | | | 1.6 (1.29-2) | < 0.001 | |
| | Age | | | 1.01 (0.99-1.03) | 0.302 | |
| Breast MAINZ | hESC | 200 | 41 | 2.22 (1.43-3.46) | < 0.001 | 0.68 (+-0.05) |
| Breast MAINZ | | 200 | 41 | | < 0.001 | 0.7 (+-0.05) |
| | hESC | | | 1.86 (1.16-3) | 0.011 | |
| | Grade | | | 1.87 (1.02-3.44) | 0.043 | |
| | Tumor Size | | | 1.13 (0.83-1.52) | 0.437 | |
| | Age | | | 1 (0.97-1.03) | 0.97 | |
| Breast UNT | hESC | 129 | 26 | 1.98 (1.21-3.25) | 0.007 | 0.69 (+-0.06) |
| Breast UNT | | 111 | 21 | | 0.01 | 0.73 (+-0.06) |
| | hESC | | | 2.08 (1.16-3.74) | 0.015 | |

| | | | | | | |
|---|---|---|---|---|---|---|
| | Grade | | | 0.87 (0.46-1.64) | 0.669 | |
| | Tumor Size | | | 1.87 (1.13-3.09) | 0.014 | |
| | Age | | | 1.03 (0.99-1.08) | 0.144 | |
| Breast TRANSBIG | hESC | 198 | 50 | 1.7 (1.2-2.42) | 0.003 | 0.66 (+-0.04) |
| Breast TRANSBIG | | 196 | 50 | | 0.004 | 0.68 (+-0.04) |
| | hESC | | | 1.52 (1.03-2.24) | 0.033 | |
| | Grade | | | 1.17 (0.73-1.86) | 0.51 | |
| | Tumor Size | | | 1.4 (1.01-1.94) | 0.041 | |
| | Age | | | 1.02 (0.98-1.06) | 0.316 | |
| Breast VDX | hESC | 344 | 118 | 1.49 (1.2-1.86) | $< 0.001$ | 0.62 (+-0.03) |
| Breast VDX | | 197 | 71 | | 0.001 | 0.66 (+-0.04) |
| | hESC | | | 1.54 (1.14-2.09) | 0.005 | |
| | Grade | | | 1.98 (1.05-3.74) | 0.036 | |
| | Age | | | 0.99 (0.97-1.01) | 0.359 | |
| Liposarcoma | hMSC | 146 | 40 | 1.53 (1.21-1.94) | $< 0.001$ | 0.66 (+-0.05) |
| Liposarcoma | | 83 | 23 | | 0.08 | 0.72 (+-0.06) |
| | hMSC | | | 1.53 (1.05-2.25) | 0.029 | |
| | Grade | | | 0 (0-Inf) | 0.997 | |
| | Tumor Size | | | 2.06 (0.65-6.54) | 0.218 | |
| Colorectal GSE14333 | hMSC | 226 | 50 | 1.76 (1.34-2.32) | $< 0.001$ | 0.68 (+-0.04) |
| Colorectal GSE14333 | | 132 | 36 | | 0.001 | 0.7 (+-0.05) |
| | hMSC | | | 1.38 (1-1.91) | 0.052 | |
| | Stage | | | 2.72 (1.31-5.62) | 0.007 | |
| Colorectal GSE17538 | hMSC | 232 | 93 | 1.45 (1.18-1.79) | $< 0.001$ | 0.62 (+-0.03) |
| Colorectal GSE17538 | | 232 | 93 | | $< 0.001$ | 0.77 (+-0.03) |
| | hMSC | | | 1.4 (1.13-1.72) | 0.002 | |
| | Stage | | | 2.79 (2.12-3.68) | $< 0.001$ | |
| Ovarian TCGA | hESC | 542 | 287 | 1.18 (1.04-1.34) | 0.009 | 0.55 (+-0.02) |
| Ovarian TCGA | | 489 | 265 | | 0.007 | 0.58 (+-0.02) |
| | hESC | | | 1.07 (0.93-1.22) | 0.331 | |
| | Debulking | | | 1.2 (0.92-1.56) | 0.171 | |
| | Stage | | | 1.45 (1.11-1.89) | 0.007 | |
| Ovarian GSE26712 | hESC | 185 | 129 | 1.38 (1.12-1.7) | 0.002 | 0.61 (+-0.03) |
| Ovarian GSE26712 | | 182 | 127 | | $< 0.001$ | 0.64 (+-0.03) |
| | hESC | | | 1.31 (1.05-1.62) | 0.016 | |
| | Debulking | | | 1.69 (1.18-2.41) | 0.004 | |
| | Stage | | | 1.21 (0.79-1.87) | 0.38 | |
| Ovarian GSE9891 | hESC | 254 | 111 | 1.44 (1.17-1.77) | $< 0.001$ | 0.64 (+-0.03) |
| Ovarian GSE9891 | | 222 | 97 | | $< 0.001$ | 0.68 (+-0.03) |
| | hESC | | | 1.27 (1-1.6) | 0.047 | |
| | Debulking | | | 1.26 (0.83-1.92) | 0.271 | |
| | Stage | | | 2.35 (1.36-4.06) | 0.002 | |
| AML GSE12417 | PB_CD34 | 163 | 103 | 1.24 (1.05-1.47) | 0.011 | 0.58 (+-0.03) |
| AML GSE12417 | | 162 | 103 | | $< 0.001$ | 0.61 (+-0.03) |
| | PB_CD34 | | | 1.15 (0.96-1.39) | 0.137 | |
| | FAB | | | 1.09 (0.95-1.25) | 0.211 | |
| | Age $> 60$ | | | 1.73 (1.17-2.56) | 0.006 | |
| AML GSE14468 | PB_CD34 | 516 | 342 | 1.18 (1.06-1.31) | 0.003 | 0.55 (+-0.02) |
| AML GSE14468 | | 457 | 290 | | $< 0.001$ | 0.61 (+-0.02) |
| | PB_CD34 | | | 1.09 (0.97-1.23) | 0.159 | |
| | Age | | | 0.98 (0.97-0.99) | $< 0.001$ | |
| | NPM1 | | | 0.66 (0.5-0.87) | 0.004 | |
| | FLT3 ITD | | | 1.76 (1.34-2.32) | $< 0.001$ | |
| DLBCL GSE4475 | PB CD34+ | 123 | 66 | 2.14 (1.43-3.2) | $< 0.001$ | 0.6 (+-0.04) |
| DLBCL GSE4475 | | 99 | 52 | | $< 0.001$ | 0.71 (+-0.04) |
| | PB CD34+ | | | 1.77 (1.11-2.82) | 0.016 | |
| | Age $> 60$ | | | 2.34 (1.28-4.27) | 0.006 | |
| | Stage | | | 1.71 (1.25-2.33) | $< 0.001$ | |
| | Extranodal sites | | | 0.95 (0.55-1.65) | 0.855 | |
| DLBCL GSE10846 | PB CD34+ | 414 | 165 | 1.55 (1.28-1.88) | $< 0.001$ | 0.6 (+-0.02) |
| DLBCL GSE10846 | | 248 | 119 | | $< 0.001$ | 0.71 (+-0.03) |
| | PB CD34+ | | | 1.41 (1.09-1.83) | 0.009 | |

| | | |
|---|---|---|
| Age $> 60$ | 1.89 (1.28-2.79) | 0.001 |
| Stage | 1.26 (1.05-1.5) | 0.013 |
| Extranodal $> 1$ | 0.97 (0.47-2) | 0.933 |
| ECOG PS | 1.55 (1.27-1.89) | $< 0.001$ |
| LDH ratio | 1.13 (1.07-1.19) | $< 0.001$ |

Supplementary Table M: Parameters used for the hESC predictor. hESC: human embryonic stem cell; hMSC: human mesenchymal stem cell; PB_CD34: CD34+ cells (peripherial blood); OS: overall survival; DMFS: distant metastasis-free survival; DFS: disease-free survival.

| Cancer Type | Genefilter | Metric | Stem Cells | Endpoint |
|---|---|---|---|---|
| AML | 2 | Pearson | PB_CD34 | OS |
| Breast Cancer | 2 | Pearson | hESC | DMFS |
| Colorectal Cancer | 2 | Pearson | hMSC | DFS |
| DLBCL | 2 | Pearson | PB_CD34 | OS |
| Liposarcoma | 2 | Pearson | hMSC | MFS |
| Lung Adenocarcinoma | 2 | Pearson | hESC | OS |
| Ovarian Cancer | 2 | Pearson | hMSC | OS |

# References


1. Barrett, T., Troup, D. B., Wilhite, S. E., Ledoux, P., Evangelista, C., Kim, I. F., Tomashevsky, M., Marshall, K. A., Phillippy, K. H., Sherman, P. M., Muertter, R. N., Holko, M., Ayanbule, O., Yefanov, A., and Soboleva, A. *Nucleic Acids Res* **39**(Database issue), 1005–1010 Jan (2011).

2. Barberi, T., Bradbury, M., Dincer, Z., Panagiotakos, G., Socci, N. D., and Studer, L. *Nat Med* **13**(5), 642–8 (2007).

3. Stirewalt, D. L., Meshinchi, S., Kopecky, K. J., Fan, W., Pogosova-Agadjanyan, E. L., Engel, J. H., Cronk, M. R., Dorcy, K. S., McQuary, A. R., Hockenbery, D., Wood, B., Heimfeld, S., and Radich, J. P. *Genes Chromosomes Cancer* **47**(1), 8–20 Jan (2008).

4. Eppert, K., Takenaka, K., Lechman, E. R., Waldron, L., Nilsson, B., van Galen, P., Metzeler, K. H., Poeppl, A., Ling, V., Beyene, J., Canty, A. J., Danska, J. S., Bohlander, S. K., Buske, C., Minden, M. D., Golub, T. R., Jurisica, I., Ebert, B. L., and Dick, J. E. *Nat Med* **17**(9), 1086–1093 Sep (2011).

5. McCusker, J. P., Phillips, J. A., González Beltrán, A., Finkelstein, A., and Krauthammer, M. *BMC Bioinformatics* **10 Suppl 10** (2009).

6. Shedden, K., Taylor, J. M., Enkemann, S. A., Tsao, M. S., Yeatman, T. J., Gerald, W. L., Eschrich, S., Jurisica, I., Giordano, T. J., Misek, D. E., Chang, A. C., Zhu, C. Q., Strumpf, D., Hanash, S., Shepherd, F. A., Ding, K., Seymour, L., Naoki, K., Pennell, N., Weir, B., Verhaak, R., Ladd-Acosta, C., Golub, T., Gruidl, M., Sharma, A., Szoke, J., Zakowski, M., Rusch, V., Kris, M., Viale, A., Motoi, N., Travis, W., Conley, B., Seshan, V. E., Meyerson, M., Kuick, R., Dobbin, K. K., Lively, T., Jacobson, J. W., and Beer, D. G. *Nat Med* **14**(8), 822–827 Aug (2008).

7. Zhu, C. Q., Ding, K., Strumpf, D., Weir, B. A., Meyerson, M., Pennell, N., Thomas, R. K., Naoki, K., Ladd-Acosta, C., Liu, N., Pintilie, M., Der, S., Seymour, L., Jurisica, I., Shepherd, F. A., and Tsao, M. S. *J Clin Oncol* **28**(29), 4417–4424 Oct (2010).

8. Gentleman, R. C., Carey, V. J., Bates, D. M., and others. *Genome Biology* **5**, R80 (2004).

9. Miller, L. D., Smeds, J., George, J., Vega, V. B., Vergara, L., Ploner, A., Pawitan, Y., Hall, P., Klaar, S., Liu, E. T., and Bergh, J. *Proc Natl Acad Sci U S A* **102**(38), 13550–13555 Sep (2005).

10. Schmidt, M., Böhm, D., von Törne, C., Steiner, E., Puhl, A., Pilch, H., Lehr, H. A., Hengstler, J. G., Kölbl, H., and Gehrmann, M. *Cancer Res* **68**(13), 5405–5413 Jul (2008).

11. Sotiriou, C., Wirapati, P., Loi, S., Harris, A., Fox, S., Smeds, J., Nordgren, H., Farmer, P., Praz, V., Haibe-Kains, B., Desmedt, C., Larsimont, D., Cardoso, F., Peterse, H., Nuyten, D., Buyse, M., Van de Vijver, M. J., Bergh, J., Piccart, M., and Delorenzi, M. *J Natl Cancer Inst* **98**(4), 262–272 Feb (2006).

12. Desmedt, C., Piette, F., Loi, S., Wang, Y., Lallemand, F., Haibe-Kains, B., Viale, G., Delorenzi, M., Zhang, Y., d'Assignies, M. S., Bergh, J., Lidereau, R., Ellis, P., Harris, A. L., Klijn, J. G., Foekens, J. A., Cardoso, F., Piccart, M. J., Buyse, M., and Sotiriou, C. *Clin Cancer Res* **13**(11), 3207–14 (2007).

13. Wang, Y., Klijn, J. G., Zhang, Y., Sieuwerts, A. M., Look, M. P., Yang, F., Talantov, D., Timmermans, M., Meijer-van Gelder, M. E., Yu, J., Jatkoe, T., Berns, E. M., Atkins, D., and Foekens, J. A. *Lancet* **365**(9460), 671–679 Feb (2005).

14. Minn, A. J., Gupta, G. P., Padua, D., Bos, P., Nguyen, D. X., Nuyten, D., Kreike, B., Zhang, Y., Wang, Y., Ishwaran, H., Foekens, J. A., van de Vijver, M., and Massagué, J. *Proc Natl Acad Sci U S A* **104**(16), 6740–6745 Apr (2007).

15. Desmedt, C., Haibe-Kains, B., Wirapati, P., Buyse, M., Larsimont, D., Bontempi, G., Delorenzi, M., Piccart, M., and Sotiriou, C. *Clin Cancer Res* **14**(16), 5158–5165 Aug (2008).

16. Barretina, J., Taylor, B. S., Banerji, S., Ramos, A. H., Lagos-Quintana, M., Decarolis, P. L., Shah, K., Socci, N. D., Weir, B. A., Ho, A., Chiang, D. Y., Reva, B., Mermel, C. H., Getz, G., Antipin, Y., Beroukhim, R., Major, J. E., Hatton, C., Nicoletti, R., Hanna, M., Sharpe, T., Fennell, T. J., Cibulskis, K., Onofrio, R. C., Saito, T., Shukla, N., Lau, C., Nelander, S., Silver, S. J., Sougnez, C., Viale, A., Winckler, W., Maki, R. G., Garraway, L. A., Lash, A., Greulich, H., Root, D. E., Sellers, W. R., Schwartz, G. K., Antonescu, C. R., Lander, E. S., Varmus, H. E., Ladanyi, M., Sander, C., Meyerson, M., and Singer, S. *Nat Genet* **42**(8), 715–21 (2010).

17. Chibon, F., Lagarde, P., Salas, S., Pérot, G., Brouste, V., Tirode, F., Lucchesi, C., de Reynies, A., Kauffmann, A., Bui, B., Terrier, P., Bonvalot, S., Le Cesne, A., Vince-Ranchère, D., Blay, J.-Y., Collin, F., Guillou, L., Leroux, A., Coindre, J.-M., and Aurias, A. *Nature medicine* **16**(7), 781–787 Jul (2010).

18. Metzeler, K. H., Hummel, M., Bloomfield, C. D., Spiekermann, K., Braess, J., Sauerland, M. C., Heinecke, A., Radmacher, M., Marcucci, G., Whitman, S. P., Maharry, K., Paschka, P., Larson, R. A., Berdel, W. E., Buchner, T., Wormann, B., Mansmann, U., Hiddemann, W., Bohlander, S. K., and Buske, C. *Blood* **112**(10), 4193–201 (2008).

19. Valk, P. J., Verhaak, R. G., Beijen, M. A., Erpelinck, C. A., Barjesteh van Waalwijk van Doorn-Khosrovani, S., Boer, J. M., Beverloo, H. B., Moorhouse, M. J., van der Spek, P. J., Löwenberg, B., and Delwel, R. *N Engl J Med* **350**(16), 1617–1628 Apr (2004).

20. Hummel, M., Bentink, S., Berger, H., Klapper, W., Wessendorf, S., Barth, T. F., Bernd, H. W., Cogliatti, S. B., Dierlamm, J., Feller, A. C., Hansmann, M. L., Haralambieva, E., Harder, L., Hasenclever, D., Kühn, M., Lenze, D., Lichter, P., Martin-Subero, J. I., Möller, P., Müller-Hermelink, H. K., Ott, G., Parwaresch, R. M., Pott, C., Rosenwald, A., Rosolowski, M., Schwaenen, C., Stürzenhofecker, B., Szczepanowski, M., Trautmann, H., Wacker, H. H., Spang, R., Loeffler, M., Trümper, L., Stein, H., Siebert, R., and Molecular Mechanisms in Malignant Lymphomas Network Project of the Deutsche Krebshilfe. *N Engl J Med* **354**(23), 2419–2430 Jun (2006).

21. Lenz, G., Wright, G., Dave, S. S., Xiao, W., Powell, J., Zhao, H., Xu, W., Tan, B., Goldschmidt, N., Iqbal, J., Vose, J., Bast, M., Fu, K., Weisenburger, D. D., Greiner, T. C., Armitage, J. O., Kyle, A., May, L., Gascoyne, R. D., Connors, J. M., Troen, G., Holte, H., Kvaloy, S., Dierickx, D., Verhoef, G., Delabie, J., Smeland, E. B., Jares, P., Martinez, A., Lopez-Guillermo, A., Montserrat, E., Campo, E., Braziel, R. M., Miller, T. P., Rimsza, L. M., Cook, J. R., Pohlman, B., Sweetenham, J., Tubbs, R. R., Fisher, R. I., Hartmann, E., Rosenwald, A., Ott, G., Muller-Hermelink, H. K., Wrench, D., Lister, T. A., Jaffe, E. S., Wilson, W. H., Chan, W. C., Staudt, L. M., and Lymphoma/Leukemia Molecular Profiling Project. *N Engl J Med* **359**(22), 2313–2323 Nov (2008).

22. Chitale, D., Gong, Y., Taylor, B. S., Broderick, S., Brennan, C., Somwar, R., Golas, B., Wang, L., Motoi, N., Szoke, J., Reinersman, J. M., Major, J., Sander, C., Seshan, V. E., Zakowski, M. F., Rusch, V., Pao, W., Gerald, W., and Ladanyi, M. *Oncogene* **28**(31), 2773–2783 Aug (2009).

23. Wu, Z., Irizarry, R. A., Gentleman, R., Martinez-Murillo, F., and Spencer, F. *Journal of the American Statistical Association* **99**(468), 909–917 (2004).

24. Jorissen, R. N., Gibbs, P., Christie, M., Prakash, S., Lipton, L., Desai, J., Kerr, D., Aaltonen, L. A., Arango, D., Kruhoffer, M., Orntoft, T. F., Andersen, C. L., Gruidl, M., Kamath, V. P., Eschrich, S., Yeatman, T. J., and Sieber, O. M. *Clin Cancer Res* **15**(24), 7642–7651 Dec (2009).

25. Guo, N. L., Wan, Y. W., Bose, S., Denvir, J., Kashon, M. L., and Andrew, M. E. *Int J Comput Biol Drug Des* **4**(1), 19–39 (2011).

26. Navab, R., Strumpf, D., Bandarchi, B., Zhu, C. Q., Pintilie, M., Ramnarine, V. R., Ibrahimov, E., Radulovich, N., Leung, L., Barczyk, M., Panchal, D., To, C., Yun, J. J., Der, S., Shepherd, F. A., Jurisica, I., and Tsao, M. S. *Proc Natl Acad Sci U S A* **108**(17), 7160–7165 Apr (2011).

27. Riester, M., Wei, W., Waldron, L., Culhane, A. C., Trippa, L., Oliva, E., Kim, S.-H., Michor, F., Huttenhower, C., Parmigiani, G., and Birrer, M. J. *Journal of the National Cancer Institute* **106**(5) May (2014).

28. Ben-Porath, I., Thomson, M. W., Carey, V. J., Ge, R., Bell, G. W., Regev, A., and Weinberg, R. A. *Nat Genet* **40**(5), 499–507 May (2008).

29. Hassan, K. A., Chen, G., Kalemkerian, G. P., Wicha, M. S., and Beer, D. G. *Clin Cancer Res* **15**(20), 6386–6390 Oct (2009).

30. Smith, J. J., Deane, N. G., Wu, F., Merchant, N. B., Zhang, B., Jiang, A., Lu, P., Johnson, J. C., Schmidt, C., Bailey, C. E., Eschrich, S., Kis, C., Levy, S., Washington, M. K., Heslin, M. J., Coffey, R. J., Yeatman, T. J., Shyr, Y., and Beauchamp, R. D. *Gastroenterology* **138**(3), 958–968 Mar (2010).

31. Cancer Genome Atlas Research Network. *Nature* **474**(7353), 609–615 Jun (2011).

32. Bonome, T., Levine, D. A., Shih, J., Randonovich, M., Pise-Masison, C. A., Bogomolniy, F., Ozbun, L., Brady, J., Barrett, J. C., Boyd, J., and Birrer, M. J. *Cancer Res* **68**(13), 5478–5486 Jul (2008).

33. Tothill, R. W., Tinker, A. V., George, J., Brown, R., Fox, S. B., Lade, S., Johnson, D. S., Trivett, M. K., Etemadmoghadam, D., Locandro, B., Traficante, N., Fereday, S., Hung, J. A., Chiew, Y. E., Haviv, I., Australian Ovarian Cancer Study Group, Gertig, D., DeFazio, A., and Bowtell, D. D. *Clin Cancer Res* **14**(16), 5198–5208 Aug (2008).

34. Wouters, B. J., Löwenberg, B., Erpelinck-Verschueren, C. A., van Putten, W. L., Valk, P. J., and Delwel, R. .

35. Haibe-Kains, B., Desmedt, C., Sotiriou, C., and Bontempi, G. *Bioinformatics* **24**(19), 2200–2208 Oct (2008).

36. van 't Veer, L. J., Dai, H., van de Vijver, M. J., He, Y. D., Hart, A. A., Mao, M., Peterse, H. L., van der Kooy, K., Marton, M. J., Witteveen, A. T., Schreiber, G. J., Kerkhoven, R. M., Roberts, C., Linsley, P. S., Bernards, R., and Friend, S. H. *Nature* **415**(6871), 530–536 Jan (2002).

37. Sotiriou, C. and Pusztai, L. *N Engl J Med* **360**(8), 790–800 Feb (2009).
utilizing established prognostic factors

[R and R/Bioconductor packages and versions]

- R version 3.1.3 (2015-03-09), `x86_64-apple-darwin13.4.0`
- Locale: `en_US.UTF-8/en_US.UTF-8/en_US.UTF-8/C/en_US.UTF-8/en_US.UTF-8`
- Base packages: base, datasets, graphics, grDevices, grid, methods, parallel, stats, utils
- Other packages: annotate 1.42.1, AnnotationDbi 1.26.1, ape 3.2, Biobase 2.24.0, BiocGenerics 0.10.0, bioDist 1.36.0, biomaRt 2.20.0, cacheSweave 0.6-1, CPE 1.4.4, DBI 0.3.1, Formula 1.2-0, gdata 2.13.3, genefilter 1.46.1, genefu 1.14.0, GenomeInfoDb 1.0.2, ggplot2 1.0.0, hgu133a.db 2.14.0, Hmisc 3.14-6, ipred 0.9-3, KernSmooth 2.23-14, lattice 0.20-30, latticeExtra 0.6-26, maphylogeny 1.0, maxstat 0.7-21, mclust 4.4, metafor 1.9-5, org.Hs.eg.db 2.14.0, penalized 0.9-45, pensim 1.2.9, plyr 1.8.1, prodlim 1.5.1, RColorBrewer 1.1-2, rmeta 2.16, rms 4.2-1, RSQLite 1.0.0, snow 0.3-13, SparseM 1.6, survC1 1.0-2, survcomp 1.14.0, survHD 0.5.0, survival 2.38-1, survivalROC 1.0.3, TTR 0.22-0, xtable 1.7-4, xts 0.9-7, zoo 1.7-11
- Loaded via a namespace (and not attached): acepack 1.3-3.3, amap 0.8-14, bitops 1.0-6, bootstrap 2014.4, class 7.3-12, cluster 2.0.1, colorspace 1.2-4, digest 0.6.8, exactRankTests 0.8-27, filehash 2.2-2, foreign 0.8-63, gtable 0.1.2, gtools 3.4.1, IRanges 1.22.10, labeling 0.3, lava 1.3, MASS 7.3-39, Matrix 1.1-5, multcomp 1.3-8, munsell 0.4.2, mvtnorm 1.0-2, nlme 3.1-120, nnet 7.3-9, polspline 1.1.9, proto 0.3-10, quantreg 5.11, Rcpp 0.11.4, RCurl 1.95-4.5, reshape2 1.4.1, rpart 4.1-9, sandwich 2.3-2, scales 0.2.4, splines 3.1.3, stashR 0.3-5, stats4 3.1.3, stringr 0.6.2, SuppDists 1.1-9.1, tcltk 3.1.3, TH.data 1.0-6, tools 3.1.3, XML 3.98-1.1
